# Dose–response relationship between physical activity and mortality in adults with noncommunicable diseases: a systematic review and meta-analysis of prospective observational studies

**DOI:** 10.1186/s12966-020-01007-5

**Published:** 2020-08-26

**Authors:** Wolfgang Geidl, Sabrina Schlesinger, Eriselda Mino, Lorena Miranda, Klaus Pfeifer

**Affiliations:** 1 Department of Sport Science and Sport, Division Exercise and Health, Friedrich-Alexander University Erlangen-Nürnberg, Gebbertstraße 123b, 91058 Erlangen, Germany; 2 Institute for Biometrics and Epidemiology, German Diabetes Center, Leibniz Center for Diabetes Research, Heinrich Heine University, Düsseldorf, Auf´m Hennekamp 65, 40225 Düsseldorf, Germany

**Keywords:** Physical activity, Health promotion, Public health, Non-communicable disease, Longevity

## Abstract

**Background:**

This study aims to investigate the relationship between post-diagnosis physical activity and mortality in patients with selected noncommunicable diseases, including breast cancer, lung cancer, type 2 diabetes, ischemic heart disease, stroke, chronic obstructive pulmonary disease (COPD), osteoarthritis, low back pain and major depressive disorder.

**Methods:**

A systematic search was conducted of PubMed, Scopus and the Web of Science from their inception to August 2018. Additionally, the search was updated in August 2019. Eligibility criteria included prospective observational studies examining the relationship between at least three physical activity categories (e.g. low, moderate, high) and all-cause mortality as the primary outcome.

**Results:**

In total, 28 studies were included in the meta-analysis: 12 for breast cancer, 6 for type 2 diabetes, 8 for ischemic heart disease and 2 for COPD. The linear meta-analysis revealed that each 10 metabolic equivalent task hours increase of physical activity per week was associated with a 22% lower mortality rate in breast cancer patients (Summary Hazard Ratio [HR], 0.78; 95% CI: 0.71, 0.86; I^2^: 90.1%), 12% in ischemic heart disease patients (HR, 0.88; 95% CI: 0.83, 0.93; I^2^: 86.5%), 30% in COPD patients (HR, 0.70; 95% CI: 0.45, 1.09; I^2^: 94%) and 4% in type 2 diabetes patients (HR, 0.96; 95% CI: 0.93, 0.99; I^2^: 71.8%). There was indication of a non-linear association with mortality risk reductions even for low levels of activity, as well as a flattening of the curve at higher levels of activity. The certainty of evidence was low for breast cancer, type 2 diabetes and ischemic heart disease but only very low for COPD.

**Conclusion:**

Higher levels of post-diagnosis physical activity are associated with lower mortality rates in breast cancer, type 2 diabetes, ischemic heart disease and COPD patients, with indication of a no-threshold and non-linear dose–response pattern.

## Background

Physical activity has been proposed as a form of treatment for people with noncommunicable diseases (NCDs) [1]. Regular physical activity positively influences symptoms and comorbidities, physical fitness and health-related quality of life in more than 25 NCDs, including osteoarthritis, type 2 diabetes (T2D), stroke and clinical depression [1]. However, it is less clear whether higher levels of physical activity in adults with NCDs also reduce mortality rates and thus lead to longer life expectancies.

The current evidence for the general population regarding physical activity and mortality is comprehensive and unambiguous. Numerous large cohort studies have consistently demonstrated an inverse relationship between physical activity levels and mortality [2]. Meta-analyses with pooled data from these studies produce similar findings [3, 4]. Compared with the lower physical activity groups, the risk of premature death was remarkably reduced in the higher physical activity groups. The meta-analysis conducted by Samitz et al. [4] revealed that per 1 hour increment of moderate-intensity physical activity per week, the relative risk of mortality was reduced by 4%. In the updated physical activity guidelines for healthy adults from the U.S. Department of Health and Human Services [5], a clear dose–response association between the volume of physical activity and mortality rates has been shown. The shape of the dose–response curve is characterized by a regressive, non-linear effect, where the greatest difference in mortality rates occurs among inactive and minimally active individuals. For higher physical activity levels, the dose–response curve flattens out. This means that the relative risk of mortality continues to decline with higher volumes of physical activity with no adverse effects on mortality, even at very high levels of physical activity [5].

In adults with NCDs, the current evidence on dose–response relations between physical activity and mortality is considerably weaker and inconsistent. For T2D, the meta-analysis conducted by Kodama et al. [6] found that an increment of one MET (metabolic equivalent task) h/day of physical activity was associated with a 9.5% relative risk reduction in all-cause mortality, thereby suggesting that post-diagnosis physical activity levels may result in similar mortality risk reductions compared to the general population. In a meta-analysis for patients with cancer, comparably beneficial associations between physical activity and mortality rates were reported by Li et al. [7]. Moore et al. [8] pooled data from six cohort studies and concluded that the longevity effects of physical activity vary according to the preexisting NCDs, with higher benefits of regular physical activity in terms of life expectancy for those with a history of cancer (7.0 y) and heart disease (6.2 y) compared to those without these diseases (3.7 y) [8]. Current evidence from the US Physical Activity Guidelines Advisory Committee [9] reported a general relationship between higher post-diagnosis physical activity and lower mortality rates in five NCDs (breast or colorectal or prostate cancer, hypertension and T2D). However, the committee found few studies that have systematically quantified the dose–response relations between physical activity levels and mortality end-points in people with preexisting NCDs. Accordingly, their report concludes that dose–response relationships cannot yet be defined for adults with NCDs [9].

Thus, the objective of this study was to conduct a systematic review and dose–response meta-analysis of physical activity and mortality in people with selected NCDs. We aimed to define the dose–response relationship between post-diagnosis physical activity and mortality rates for nine NCDs with a high global burden of disease [10], including low back pain, T2D, osteoarthritis, depressive disorder, chronic obstructive pulmonary disease (COPD), breast cancer, lung cancer, stroke and ischemic heart disease (IHD).

## Methods

The method for this systematic review and meta-analysis was predefined in a published study protocol [11], and registered at PROSPERO – the International Prospective Register of Systematic Reviews (registration number: CRD42018103357; available online at https://www.crd.york.ac.uk/prospero/display_record.php?RecordID=103357). This systematic review and meta-analysis is reported in compliance with the Preferred Reporting Items for Systematic Reviews and Meta-Analyses (PRISMA) Statement for Reporting Systematic Reviews and Meta-Analysis (see Supplementary file 1) [12].

### Search and data sources

A systematic search was conducted on PubMed, Scopus and the Web of Science from their inception to August 2018. This search was followed by a hand-search of the citations in the detected articles. The search was updated in August 2019 by using the forward citation search in Google Scholar for the articles that qualified for inclusion (see Supplementary file 2).

### Study selection

The eligibility criteria required the population to consist of adults with a physician-confirmed or self-reported diagnosis of one of the nine NCDs (osteoarthritis, low back pain, depressive disorder, IHD, T2D, stroke, COPD, lung cancer or breast cancer). Studies that investigated the association between physical activity and all-cause mortality as the primary outcome or any other indication-specific mortality as a primary or secondary outcome were included. To calculate the dose–response meta-analysis, at least three categories of the exposure (i.e. physical activity) had to be reported in the original study. The eligible study design was that of a prospective observational nature. Non-English-language records, studies conducted on non-human subjects and duplicate data sets were not considered. No limit on publication year was imposed.

First, the literature identified through the electronic search was primarily assessed for eligibility by inspecting the titles and abstracts. We decided to divide the literature between three reviewers because of the large number of hits. Two additional reviewers were appointed to ensure the quality of the first screening process. In the second step, the full texts of the qualified studies were retrieved and critically evaluated for their final inclusion in the data collection process. The three reviewers independently assessed the articles for eligibility, and any discrepancies were resolved by discussions and when necessary, by adjudication from another reviewer.

### Data collection and items

The following details were extracted from the included publications: first author, year of publication, study name, design, country, mean follow-up time, total sample size, age, sex, mortality cases in total and per physical activity category, exposure categories, diagnosis and mortality ascertainment, relative risks and corresponding 95% CIs of the multivariate-adjusted models. Thirteen authors of the selected studies were contacted for additional data on physical activity. However the original data from two authors did not allow for an estimation of physical activity levels in MET-h/week (meaning that these studies were excluded), and two authors provided information on physical activity dosage [13, 14].

### Risk of bias in individual studies

The Cochrane tool for assessing the “Risk Of Bias In Non-randomised Studies - of Interventions” (ROBINS-I) was used to estimate the risk of bias and endorse conclusions closer to the truth [15]. The tool includes seven domains that lead to the risk of bias. These domains are due to 1) confounding, 2) selection of participants, 3) exposure assessment, 4) misclassification during follow-up, 5) missing data, 6) measurement of the outcome, and 7) selective reporting of results. The domains 1, 2 and 3 are directly considered in rating the certainty of evidence. The included studies were independently evaluated by two assessors (EM, LM). Any inconsistencies in the evaluations were documented and then discussed with a third member of the research team (WG) and resolved by mutual agreement.

### Statistical analysis

The meta-analysis was performed using Stata statistical software (Version 15, StataCorp, College Station, TX, US). We pooled aggregated data using the random effects meta-regression model, as suggested by DerSimonian and Laird [16], assuming random variance of the true effect of physical activity among studies, especially due to diversity in assessment methods. For studies that reported results from one cohort in stratified estimates (e.g. separately for men and women), a fixed effect model was used to combine the effects for the whole cohort and include it in the meta-analysis. We conducted the linear dose–response association between physical activity per 10 MET-h/week and all-cause mortality via the method used by Greenland and Longnecker and presented via forest plots [17, 18]. For this analysis, the number of cases and person-years, the quantification of the exposure and RRs with the corresponding 95% CIs of at least three categories were needed. If information was missing, the distributions of cases and person-years were estimated using the total number of cases and the total number of participants plus the follow-up period, as previously described [19]. If the lowest category was not used as a reference, the reported risk estimates were recalculated using Orsini et al.’s [20] method to ensure comparability. The data on the volume of physical activity were converted into a unit of MET-h/week. If a study reported the exposure categories as ranges, then for each category, the midpoint between the lower and upper limit was calculated. For open categories, we assumed that the width was the same as the adjacent category. A potential non-linear association was evaluated using a restricted cubic spline model with three knots at the 10th, 50th and 90th percentile of frequency of the exposure [18]. The indication of nonlinearity was tested using a likelihood ratio test.

The heterogeneity was described using the measure of inconsistency (I^2^), and tau^2^ was used to measure the variance between the included studies [21]. Subgroup analysis and meta-regression were performed to explore the heterogeneity across studies. The analyses were stratified by demographic variables (age, geographic area), follow-up duration (< 10 and ≥ 10 years), death cases (< 100, 100–500 and ≥ 500), method of physical activity assessment (questionnaire and interview), risk of bias (moderate and serious) and additional disease-specific relevant factors (e.g. menopausal status in breast cancer). Publication bias was investigated through various visual and statistical tools, including funnel plots and Egger’s test for small-study effects, where asymmetry with a significance level of *p* < 0.1 suggests publication bias [22, 23].

### Certainty of evidence

We used the Grading of Recommendations, Assessment, Development and Evaluation (GRADE) approach to assess the certainty of evidence [24]. GRADE includes four certainty categories (very low, low, moderate or high) and we worked with GRADE profiler (GRADEPRO) to present the overall certainty of evidence for the outcome of interest based on the included studies [25]. One reviewer assessed the certainty of evidence and a second reviewer revised the certainty assessments as necessary. Due to the inherent limitations in observational studies, certainty of evidence starts at low and can be further downgraded (based on risk of bias, imprecision, inconsistency of results, indirectness of evidence and publication bias) or upgraded (based on large magnitude of effect, a dose-response gradient, or opposing residual confounding) [26]. Following the GRADE recommendations for informative statements to communicate the findings we developed summary of findings tables and included plain language summaries based on effect magnitude and certainty of evidence in the result section [27].

## Results

**Fig. 1:**
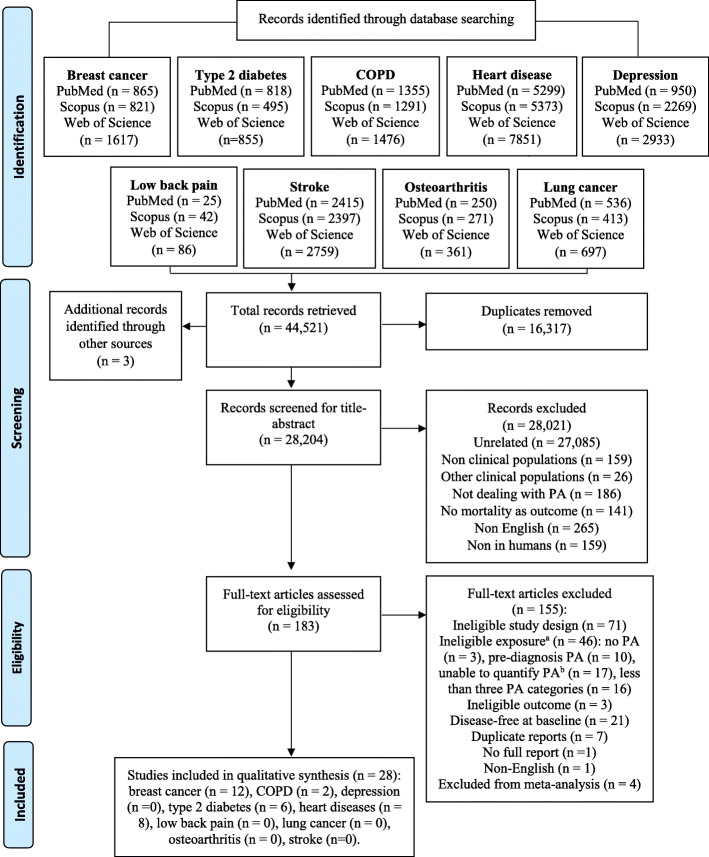
PRISMA flow chart. Explanations. a. Studies with *ineligible exposure* did not directly measure the physical activity but another facet of movement behaviour mostly sedentary time; b. For studies excluded based on the *unability to quantify physical activity*, it was impossible to quantify physical activity because data presented in the paper do not allow for the transformation of the activity categories in MET-h/week (e.g. when the question was “compared to other men of your age do you intend to walk slower, faster or about the same?”)

The systematic database search yielded 44,518 publications in total. Three additional studies were identified from the reference lists. Full texts were retrieved and screened for 183 articles with the potential for inclusion. Twenty-eight studies satisfied the inclusion criteria for only four out of the nine NCDs: breast cancer (*n* = 12), T2D (*n* = 6), IHD (*n* = 8) and COPD (*n* = 2) (see Fig. 1 for a detailed flow diagram).

### Study characteristics

The 28 included studies were all published during the past two decades and based in numerous countries throughout the world. Out of these, 25 studies were prospective cohort studies, 2 prospective follow-ups to case-control studies and 3 follow-up studies of RCTs [13, 28, 29]. The sample sizes varied considerably from 435 [30] to 15,645 [31], with a total of 27,248 participants diagnosed with breast cancer, 32,221 with T2D, 4784 with COPD and 42,027 with IHD.

The follow-up duration ranged from 3.3 years [32] to 18.4 years [33]. A summary of the main characteristics of the cohorts is displayed in Table 1. All-cause mortality was reported as the primary outcome in all included studies. Other reported outcomes were breast cancer mortality, recurrence and new primary events, cardiovascular disease mortality, IHD mortality and respiratory mortality. All exposure assessments of post-diagnosis physical activity were based on self- or interviewer-administered questionnaires. The time from diagnosis to physical activity measurement varied from three to 6 months post-diagnosis [48]. The longest follow-up was 14 years [53]. Detailed information on the measurement instruments for physical activity assessment can be found in Supplementary file 3. Exposure categories were presented as the volume of physical activity in MET-h/week [14, 28–32, 34–42, 44–46, 52], calorie expenditure [49], duration of physical activity [43], frequency of physical activity [51], and nominal categories [13, 33, 47, 48, 50, 53].

**Table 1:** Main characteristics of the included studies

Publication, study name, study design, country	Sample size/ No of deaths	Follow-up	Diagnosis and mortality ascertainment	Physical activity measurement	Exposure category	Risk estimates	Confounders	Risk of bias assessment
**Breast cancer**								
Ammitzbøll 2016 [34], DCHS, Denmark, prospective cohort study, USA	*n* = 959 144 cases	Median (IQR), y = 10 (7)	Cancer registry; Central Population Register.	Questionnaire	*MET-h/week:* 0 0–41 > 41–63 > 63–97 > 97–379	1.5 >(0.83–2.72) 1 0.75 (0.45–1.24) 0.83 (0.49–1.40) 0.75 (0.42–1.33)	BMI, baseline alcohol, smoking status, education, comorbidity, nodal status, operation type, chemotherapy, recreational and household PA.	Serious
Bao 2015 [35], SBCSS, prospective cohort study, China	*n* = 518 128 cases	Median (range), y = 9.1 (0.6–11.8)	Shanghai Cancer Registry; Shanghai Vital Statistics Registry.	Questionnaire	*MET-h/week:* 0 < 7.6 ≥ 7.6	1 0.79 (0.5–1.27) 0.61 (0.41–0.91)	Age at diagnosis, BMI at baseline, education, marital status, menopausal status, Charlson comorbidity index, chemotherapy, radiotherapy, tumor-node metastasis stage, soy protein intake, tea consumption at baseline.	Moderate
Bertram 2011 [28], WHEL, follow-up study of RCT, USA	*n* = 2361 195 cases	Median (range), y = 7.1 (1.0–10.8)	Medical records and death certificates, telephone interviews, confirmation obtained for > 95% of participants.	Questionnaire	*MET-h/week:* 0–2.5 2.5–7.5 7.5–14.9 14.9–24.7 24.7–107	1 1.01 (0.66–1.55) 0.85 (0.53- 1.35) 0.75 (0.46–1.23) 0.47 (0.26- 0.84)	Age and BMI at randomization, race, fruit and vegetable consumption, menopausal status, tumor type, tumor grade, tumor stage, anti-estrogen use, clinical site, time from diagnosis to randomization, hot flashes, and study group.	Moderate
Bradshaw 2014 [36], LIBCSP, prospective cohort follow-up of case-control study, USA	*n* = 1423 420 cases	Median (range), y = 12.7 (0.23–13.42)	Physician confirmed diagnosis; National Death Index.	Interviewer-administered questionnaire	0 0.1–9 > 9	1 0.43 (0.20–0.83) 0.32 (0.23–0.47)	Age, pre-diagnosis BMI, chemotherapy treatment, tumor size, missing PA data.	Serious
Chen 2011 [37], SCR, prospective cohort study, China	*n* = 4826 436 cases	Median, y = 4.3	Population-based Shanghai Cancer Registry; Annual linkage with the Shanghai Vital Statistics database.	Interview	*MET-h/week:* 0 < 8.3 > 8.3	1 0.81 (0.63–1.05) 0.65 (0.51- 0.84)	Date of birth, BMI at baseline, waist-to-hip ratio at baseline, menopausal status, income, education, quality of life, cruciferous vegetable intake, soy protein intake, tea consumption, chemotherapy, radiotherapy, tamoxifen use, tumor-node metastasis status, estrogen progesterone receptor status.	Moderate
de Glas 2014 [30], TEAM-L, prospective cohort study, Netherlands	*n* = 435 58 cases	Followed until 2012 (from 2004 to 5)	Histologically/cytologically confirmed diagnosis.	Questionnaire	*MET-h/week:* 0–21 21.1–40 40.1–65.5 65.6–258	1 0.43 (0.19- 0.94) 0.60 (0.29- 1.24) 0.57 (0.26- 1.40)	Age at 1 year after diagnosis, number of comorbidities, tumor stage, node stage, BMI, and chemotherapy.	Moderate
Holick 2008 [38], CWLS, prospective cohort follow-up of case-control study, USA	*n* = 4482 412 cases	Mean, y = 5.5 ± 1.1	Self-reported and state cancer registries; National Death Index.	Questionnaire	*MET-h/week:* < 2.8 2.8–7.9 8.0–20.9 ≥ 21.0	1 0.58 (0.45–0.76) 0.53 (0.40- 0.69) 0.44 (0.32- 0.60)	Age at diagnosis, stage of disease at diagnosis, state of residence, interval between diagnosis and PA assessment, BMI, post-diagnosis menopausal status, post-diagnosis hormone therapy use, total energy intake year before enrollment in the CWLS, education level at diagnosis, family history of breast cancer at diagnosis, and initial treatment modality (radiation, chemotherapy, tamoxifen).	Serious
Holmes 2005 [39], NHS, prospective cohort study, USA	*n* = 2987 463 cases	Median, y = 8	Self-reported and confirmed from medical records and pathology reports, family, postal authorities; National Death Index.	Questionnaire	*MET-h/week:* < 3 3–8.9 9–14.9 15–23.9 ≥24	1 0.71 (0.56- 0.89) 0.59 (0.41- 0.84) 0.56 (0.41- 0.77) 0.65 (0.48- 0.88)	Age, interval between diagnosis and PA assessment, smoking status, BMI, menopausal status and hormone therapy use, age at first birth and parity, oral contraceptive use, energy intake, energy-adjusted protein intake, disease stage, radiation treatment, chemotherapy, and tamoxifen treatment.	Serious
Irwin 2008 [40], HEAL, prospective cohort study, USA	n post-diagnosis = 688 53 cases	Median (range), y = 6 (5–8)	Surveillance, Epidemiology, and End Results registries.	Questionnaire	*MET-h/week:* 0 > 0–8.9 ≥ 9	1 0.36 (0.17–0.73) 0.33 (0.15–0.73)	Age, race, disease stage, initial treatment, tamoxifen use, BMI, and fruit/vegetable servings per day.	Serious
Irwin 2011 [32], WHI, prospective cohort study, USA	*n* = 2910 186 cases	Mean (SD), y = 3.3 (1.8)	Physician-confirmed diagnosis, clinical center follow-up of participants and surrogates; National Death Index.	Questionnaire	*MET-h/week:* 0 > 0–3.0 3.1–8.9 ≥ 9	1 0.42 (0.21–0.82) 0.72 (0.48- 1.07) 0.54 (0.38-0.79)	Age, stage, estrogen receptor, progesterone receptor, grade, human epidermal growth factor receptor 2, ethnicity, study arm, previous therapy use, time from diagnosis to PA assessment, BMI, diabetes, alcohol consumption, smoking, total calories, percentage calories from fat, servings of fruit and vegetables.	Moderate
Maliniak 2018 [41], CPS-II NC, prospective cohort study, USA	*n* = 3689 185 cases	Median (IQR) = 7.5 (5.8)	Self-reported diagnosis of breast cancer; National Death Index. Both verified through medical records or state cancer registries.	Questionnaire	*MET-h/week:* < 3.5 3.5 - < 8.75 8.75 - < 17.5 > 17.5 < 3.5 3.5 - < 8.75 8.75 - < 17.5 > 17.5	*Age 45–64 years:* 1.36 (0.89–2.07) 1 0.67 (0.44–1.02) 0.56 (0.37–0.83) *Age 65–92 years:* 1.34 (1.14–1.58) 1 0.81 (0.67–0.99) 0.74 (0.61–0.90)	Age at diagnosis, race, calendar year of diagnosis, post-diagnosis BMI, Surveillance, Epidemiology, and End Results summary stage at diagnosis, post-diagnosis number of co-morbidities, post-diagnosis use of hormone replacement therapy, post-diagnosis alcohol intake, and post-diagnosis other cancer diagnosis, smoking status.	Serious
Sternfeld 2009 [42], LACE, prospective cohort study, USA	*n* = 1970 187 cases	Mean (SD), mo = 87 (18)	Kaiser Permanente Northern California Cancer Registry, Utah Cancer Registry; death certificates.	Questionnaire	*MET-h/week:* < 29 29- < 44 44- < 62 ≥ 62	1 0.89 (0.59–1.33) 0.82 (0.54–1.25) 0.76 (0.48–1.19)	Age, BMI, number of positive nodes, stage, weight at 18 years, type of treatment, type of surgery, education level, smoking status.	Serious
**Type 2 diabetes**								
Glenn 2015 [31], SCCS, prospective cohort study, USA	*n* = 15,645/ m-f 2370 cases	Median (range), y = 6.2 (0.01–9.8)	Self-reported diagnosis; Social Security Administration vital status service for epidemiologic researchers and the National Death Index.	Questionnaire	*MET-h/day:* < 6.9 6.9–14.1 14.2–24.8 > 24.9	1 0.77 (0.69–0.86) 0.66 (0.58–0.74) 0.64 (0.57–0.73)	Age, sex, race, BMI, income, education, comorbidities (hypertension, high cholesterol, myocardial infarction, stroke), smoking, insulin use, time since diagnosis, sedentary time.	Serious
Gregg 2003 [43], NHIS, prospective cohort study, USA	*n* = 2896/ m-f 671 cases	y = 8	Self-reported diagnosis; National Death Index.	Interview	*h/week:* 0 > 0–1.9 ≥ 2	1 0.95 (0.77-1.17). 0.71 (0.59-0.87)	Age, BMI, sex, race, self-rated health, smoking, weight loss approaches, hospitalizations, hypertension, use of antihypertensive medications, physician visits, limitations caused by cancer and CVD, functional limitations.	Serious
Hu 2004 [33], six independent population surveys, prospective cohort study, Finland	*n* = 3316/ m-f 1410 cases	Mean, y = 18.5	Self-reported, hospital discharge diagnosis, or drug-treated cases in the Drug Registry; Statistics Finland.	Questionnaire	Low Moderate High	1 0.95 (0.81- 1.12) 0.96 (0.80- 1.15)	Age, sex and study year, BMI, systolic blood pressure, cholesterol, smoking, occupational PA, commuting PA; individuals with comorbidities, severe disease or disability at baseline, and who died the first two years of follow-up were excluded.	Serious
Sluik 2012 [44], EPIC, prospective cohort study, 10 European Countries	*n* = 5859/ m-f 755 case	Median, y = 9.4	Self-reported diabetes confirmed from a physician, or use of medication, or self-reported confirmation during follow-up, or diabetes registries, or HbA1c level > 6% at baseline; Linkages with local, regional, or central cancer registries, boards of health, or death indices.	Questionnaire	*MET-h/week:* < 45 45–74 75–113 > 113	1 0.85 (0.70–1.04) 0.80 (0.64–0.99) 0.73 (0.57–0.93)	Age, sex, study center, diabetes medication, disease duration, myocardial infarction, stroke, cancer, alcohol consumption, smoking behavior, education, energy intake, scores for dietary patterns.	Serious
Sone 2013 [45], JDCS, prospective cohort study, Japan	*n* = 1702/ m-f 69 cases	Median, y = 8.05	HbA1c levels ≥ 6.5% (51 mmol/mol) referring to the Japan Diabetes Society; annual reports form.	Questionnaire	*MET-h/week:* ≤ 3.7 3.8–15.3 ≥ 15.4	1 0.88 (0.47–1.64) 0.47 (0.22–0.99)	Age, sex, BMI, diabetes duration, smoking, energy/ethanol intake, dietary fiber, saturated fatty acid, type of occupation, HbA1c, systolic blood pressure, LDL-cholesterol, HDL-cholesterol, triacylglycerol, treatment (insulin, oral hypoglycaemic agents, antihypertensive agents or lipid-lowering agents).	Serious
Tanasescu 2003 [46], HPFS, prospective cohort study, USA	*n* = 2803/ m 355 cases	y = 14	Self-reported physician’s diagnosis confirmed from diagnostic criteria from the National Diabetes Data Group; next of kin, work associates, postal authorities, and National Death Index.	Questionnaire	*MET-h/week:* 0–5.1 5.2–12.0 12.1–21.7 21.8–37.1 ≥37.2	1 0.88 (0.64–1.21) 0.64 (0.45–0.91) 0.64 (0.45–0.90) 0.65 (0.45–0.93)	BMI, alcohol intake, smoking status, family history of myocardial infarction, use of vitamin E supplements, diabetes duration, diabetes medication, dietary intake of trans fat, saturated fat, fiber, and folate, history of angina and coronary artery bypass graft (CABG), hypertension at baseline, high serum cholesterol at baseline.	Moderate
**Chronic obstructive pulmonary disease**								
Cheng 2018 [14], HSE and SES, prospective cohort study, England and Scotland	*n* = 2398/ m-f 571 cases	Mean (SD), y = 8.5 (3.9)	Confirmed by spirometry according to GOLD criteria; National Health Service mortality data.	Questionnaire	*MET-h/week:* 0 < 3.75 3.75- < 7.5 ≥ 7.5	1 0.86 (0.67–1.10) 0.75 (0.56–1) 0.56 (0.45–0.69)	Age, gender, BMI, COPD severity, history of CVD, cancer and diabetes, self-reported longstanding illness, smoking status, alcohol consumption, education.	Serious
Garcia-Aymerich 2006 [47], CCHS, prospective cohort study, Denmark	*n* = 2386/ m-f 1425 cases	Mean (SD) = 12 (5.9)	Confirmed by a fixed FEV1/FVC ratio test; Danish National Board of Health.	Questionnaire	Very low Low Moderate High	1 1.04 (0.84–1.27) 0.73 (0.61–0.86) 0.72 (0.59–0.86)	Age, gender, BMI, income, smoking status, glucose, systolic blood pressure FEV_1_, IHD, myocardial infarction, stroke, asthma, sputum, asthma and smoking interaction.	Serious
**Ischemic heart diseases**								
Gerber 2011 [48], ISFAMI, prospective cohort study, Israel	*n* = 1521/ m-f 427 cases	Median (IQR), y = 13.2 (12.0–13.5)	Index hospitalization; Israeli Population Registry, death certificates, hospital charts, family physicians and members.	Questionnaire	None Irregularly Regularly	1 0.71 (0.54–0.95) 0.56 (0.42–0.74)	Age, gender, hypertension, diabetes, dyslipidemia, smoking, obesity, chronic IHD, comorbidity index, Killip class, self-rated health, thrombolytic therapy, CABG, PTCA within 45 days, education, income, pre-MI employment, living with a steady partner, recurrent MI (and unstable angina pectoris, heart failure, CABG and PTCA) and cardiac rehabilitation during follow-up.	Moderate
Janssen 2006 [49], CHS, prospective cohort study, USA	*n* = 1045/ m-f 489 cases	y = 9	Self-reported IHD confirmed from medications, medical records, clinical examinations; reviews of obituaries, medical records, death certificates, and the U.S. Health Care Financing Administration health care utilization database.	Interview	*EE (kcal/week)* < 500 500–999 1000–1999 2000–2999 ≥ 3000	1 0.87 (0.68–1.26) 0.77 (0.59–0.99) 0.54 (0.36–0.81) 0.63 (0.44–0.91)	Age, sex, race, smoking, alcohol, socioeconomic status, adiposity, prevalent disease (diabetes, lung disease, cancer, hypertension, stroke, congestive heart failure), and type of IHD (angina, MI, coronary revascularization).	Moderate
Lahtinen 2017 [13], ARTEMIS, follow-up study of RCT, Finland	*n* = 1746/ m-f 147 cases	Median (IQR), mo = 54 (41–69)	Angiographically confirmed IHD with coronary stenosis > 50% of ≥ 1 coronary arteries; national death registries, mailing, telephone calls to family, electronic patient records.	Questionnaire	Inactive Irregularly active Active Highly active	4.6 (2.1–10.0) 2.4 (1.1–5.3) 2.0 (0.9–4.3) 1	Age, gender, BMI at baseline, diabetes mellitus, history of MI, left ventricular ejection fraction, Canadian Cardiovascular Society grading for angina pectoris, cardiovascular event at 2-years follow-up, smoking status and alcohol consumption at 2 years.	Moderate
Moholdt 2017 [50], HUNT, prospective cohort study, Norway	*n* = 6493/ m-f 3818 cases	Median (IQR), y = 12.5 (14.1)	Self-reported IHD; National Cause of Death Registry in Norway.	Questionnaire	Inactive Low Recommended High	1 0.85 (0.79–0.92) 0.81 (0.72–0.90) 0.82 (0.70–0.95)	Age, examination year, smoking status, diabetes mellitus, alcohol consumption, hypertension, health status, PA for BMI, BMI for physical activity (stratified by sex).	Moderate
Mons 2014 [51], KAROLA, prospective cohort study, Germany	*n* = 1038/ m-f	Mean (SD), y = 8.1 (3.1)	Physician-confirmed diagnosis; death certificates.	Questionnaire	*Frequency/strenuous PA:* Rarely/never 1-4x/month 2-4x/week 5-6x/week Daily	3.81 (2.17–6.70) 1.74 (0.59–3.21) 1 1.69 (0.81–3.50) 1.77 (0.90–3.47)	Age, gender, BMI, education, study site, employment status, cotinine-validated smoking status, self-reported poor health, history of MI, diabetes mellitus, hypertension, left ventricular function, number of affected vessels.	Serious
Stewart 2017 [29], STABILITY, follow-up study of RCT, Global (39 countries)	*n* = 15,467/ m-f 1081 cases	Median (IQR), y = 3.79 (0.31)	IHD confirmed from coronary angiography.	Questionnaire	*Median (IQR), MET-h/w:* 14.0 ± 12.0 40.0 ± 14.0 90.0 ± 52.0	1 0.75 (0.65–0.87) 0.70 (0.60–0.82)	Age, gender, BMI, randomized treatment, systolic blood pressure, hypertension, geographic region for final reporting, prior myocardial infarction (MI), prior coronary revascularization percutaneous coronary intervention or coronary artery bypass graft, IHD, diabetes mellitus, smoking status, polyvascular disease, significant renal dysfunction, hemoglobin, white blood cell count, low-density lipoprotein cholesterol, high-density lipoprotein cholesterol, triglycerides, estimated glomerular filtration rate according to the Chronic Kidney Disease-Epidemiology research group calculator, congestive heart failure.	Serious
Tian 2017 [52], CKB, prospective cohort study, China	n (IHD) = 13,936 / m-f 1442 cases	y = 7	Physician-diagnosed hypertension or CVD (stroke, transient ischemic attack, ischemic heart disease); China CDC’s Disease Surveillance Points system, local residential and health insurance records, active confirmation from street committee or village administrators.	Interviewer-administered questionnaire	*MET-h/d:* < 8.91 8.91–16.53 ≥ 16.53	1 (0.93 - 1.08) 0.80 (0.73–0.88) 0.59 (0.50–0.70)	Smoking status, alcohol consumption, fruit consumption, dairy products, meat and preserved vegetables, education, income, survey season, diabetes status, family history of CVD, CVD medication, poor health status (Analysis stratified by age-at-risk, gender, region, baseline CVD status)	Serious
Wannamethee 2000 [53], BRHS, prospective cohort study, Great Britain	*n* = 772/ m 131 cases	Mean, y = 5	Self-reported physician-diagnosed IHD; National Health Service registers in Southport and Edinburgh.	Interviewer-administered questionnaire	Inactive/occasional Light Moderate Moderate to vigorous	1 0.42 (0.25–0.71) 0.47 (0.24–0.92) 0.63 (0.39–1.03)	Age, smoking, social class, self-rated health status, diabetes mellitus, history of MI and stroke.	Moderate

*DCHS* Diet, Cancer, and Health study, *IQR* Interquartile range, *BMI* body mass index, *PA* physical activity, *CVD* cardiovasular diseases, *IHD* ischemic heart diseases, *COPD* chronic obstructive pulmonary disease, *SBCSS* Shanghai Breast Cancer Survival Study, *WHEL* Women’s Healthy Eating and Living study, *LIBCSP* Long Island Breast Cancer Study, *LIBCSP* Long Island Breast Cancer Study Project, *SCR* Shanghai Cancer Registry, *TEAM-L* Tamoxifen Exemestane Adjuvant Multicenter Lifestyle, *CWLS* Collaborative Women’s Longevity Study, *NHS* Nurses’ Health Study, *HEAL* Health, Eating, Activity, and Lifestyle study, *WHI* Women’s Health Initiative study, *CPS-II NC* Cancer Prevention Study-II Nutrition Cohort, *LACE* Life After Cancer Epidemiology study, *SCCS* Southern Community Cohort Study, *NHIS* National Health Interview Survey, *EPIC* European Prospective Investigation Into Cancer and Nutrition, *JDCS* Japan Diabetes Complications Study, *HPFS* Health Professionals’ Follow-up Study, *HSE* Health Survey for England, *SES* Scottish Health Survey, *CCHS* Copenhagen City Heart Study, *ISFAMI* Israel Study of First Acute Myocardial Infarction, *CABG* coronary artery bypass graft, *PTCA* percutaneous transluminal coronary angioplasty, *CHS* Cardiovascular Health Study, *ARTEMIS* Innovation to Reduce Cardiovascular Complications of Diabetes at the Intersection, *HUNT* Nord-Trøndelag Health study, *CKB* China Kadoorie Biobank study, *BRHS* British Regional Heart Study

### Risk of bias in included studies

**Fig. 2:**
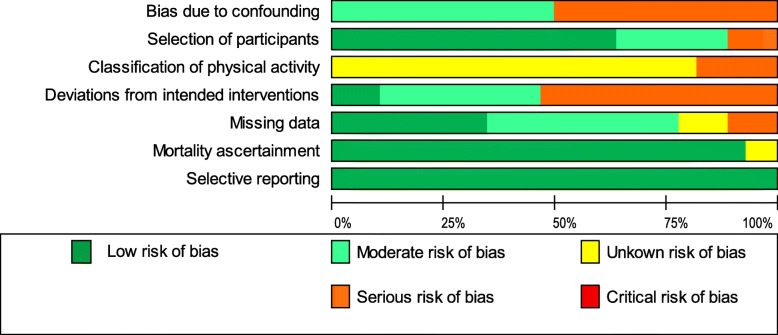
Risk of bias graph

In terms of the seven domains of ROBINS-I, no study had a low risk of bias. From the 28 publications assessed, eleven were evaluated to have a moderate risk of bias [13, 28, 30, 32, 35, 37, 46, 48–50, 53], and the remaining studies had a serious risk of bias. The main domains that introduced bias were 1) the confounding domain (mainly due to the lack of adjusting for potential confounders such as the level of physical activity before diagnosis) and 2) the domain of deviations from intended interventions. This point refers to the uncertainty as to whether the physical activity exposure measured at a given time in the observational studies is representative for the habitual activity levels of the individuals. It is important to note that all studies used validated measurement instruments (see Supplementary file 3) but due to the assessment of physical activity based on self-reports, a potential misclassification of physical activity could not be excluded. Therefore, 82% of the studies were rated with an unknown risk of bias in the domain of the classification of physical activity. The risk of bias for each domain in the 28 studies is shown in Fig. 2. In addition, Table 1 contains the final risk of bias evaluation across the studies, and Supplementary file 4 includes the detailed results of the risk of bias assessment. The overall certainty of evidence for the reduction of all-cause mortality germane to post-diagnosis physical activity is presented in Table 2.

**Table 2:** Summary of findings

Post-diagnosis physical activity and all-cause mortality for patients with breast cancer, COPD, type 2 diabetes and IHD				
Outcome	Relative effect per 10 MET-h/week (95% CI)	№ of participants (studies)	Certainty of the evidence (GRADE)	Informative statements
All-casue mortality for breast cancer	**HR 0.78** (0.71 to 0.86)	27,248 (12 observational studies)	⨁⨁◯◯ LOW ^a,b,c^	The evidence suggests post-diagnosis physical activity results in a slight reduction in all-cause mortality for individuals with breast cancer.
All-cause mortality for COPD	**HR 0.70** (0.45 to 1.09)	4784 (2 observational studies)	⨁◯◯◯ VERY LOW ^b,c,d,e,f^	The evidence is very uncertain about the effect of post-diagnosis physical activity on all-cause mortality for individuals with COPD.
All-cause mortality for IHD	**HR 0.88** (0.83 to 0.93)	42,027 (8 observational studies)	⨁⨁◯◯ LOW ^b,c,g^	The evidence suggests post-diagnosis physical activity results in a slight reduction in all-cause mortality for individuals with IHD.
All-cause mortality for type 2 diabetes	**HR 0.96** (0.93 to 0.99)	32,221 (6 observational studies)	⨁⨁◯◯ LOW ^a,b,c^	The evidence suggests post-diagnosis physical activity results in a slight reduction in all-cause mortality for individuals with type 2 diabetes.

Explanations:

^a^Downgraded by two levels since five studies were judged as serious risk of bias regarding confounding or selection bias based on ROBINS-I

^b^Downgraded by one level because although exposure was assessed in all studies using validated questionnaires, there were differences in the assessment and calculation of physical activity levels

^c^Upgraded by one level due to the dose-response gradient

^d^Downgraded by two levels since two studies were judged as serious risk of bias regarding confounding or selection bias based on ROBINS-I

^e^Downgraded by one level because the 95% CI includes the null value (HR = 1.0) and includes important benefits HR < 0.75

^f^Downgraded by one level because publication bias could not be assessed due to limited number of studies (< 5 studies)

^g^Downgraded by two levels since three studies were judged as serious risk of bias regarding confounding or selection bias based on ROBINS-I

### Post-diagnosis physical activity and all-cause mortality

**Fig. 3:**
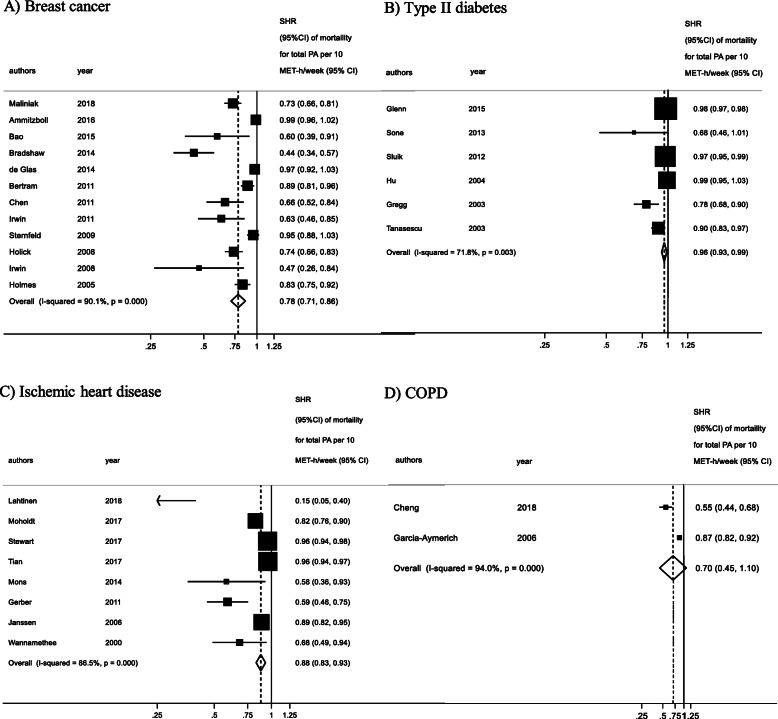
Linear dose–response meta-analysis for the association between post-diagnosis physical activity and all-cause mortality

We examined the relationship between post-diagnosis physical activity and all-cause mortality in breast cancer, T2D, IHD and COPD populations. The results of the linear dose–response meta-analyses are presented in Fig. 3. Physical activity was associated with lower mortality rates in persons with breast cancer, T2D, COPD and IHD. For every 10 MET-h increase of physical activity per week, the summary hazard ratio (SHR) decreased by 22% in people with breast cancer (HR, 0.78; 95% CI: 0.71, 0.86; *n* = 12) [28, 30, 32, 34–42], by 12% in people with IHD (HR, 0.88; 95% CI: 0.83, 0.93; *n* = 8) [13, 29, 48–53], and by 30% in people with COPD (HR, 0.70; 95% CI: 0.45, 1.09; *n* = 2) [14, 47]. The mortality rates in people with T2D reduced by 4% for every 10 MET-h/week (HR, 0.96; 95% CI: 0.93, 0.99; *n* = 6) [31, 33, 43–46]. Ten MET-hours/week is equivalent to 180 min of walking or 86 min of running [54]. These findings are based on a very low certainty of evidence for COPD, and low certainty of evidence for breast cancer, IHD, and T2D (see Table 2 and the GRADE evidence profile in Supplementary file 5).

There was evidence of high heterogeneity between the included studies for all the target groups, specifically breast cancer (I^2^ = 90.1%), T2D (I^2^ = 72.7%), IHD (I^2^ = 86.5%) and COPD (I^2^ = 94.0%). The subgroup analysis for breast cancer (Supplementary file 7, Table S7.1) highlighted that there are no differences regarding menopausal status, assessment of physical activity, geographical area, age or number of cases; however, the subgroup difference is statistically significant (*p* = 0.018) for the follow-up variable only, meaning that the length of follow up can modify the observed associations between physical activity and mortality. None of the other included variables (age, geographic area, death cases, method of physical activity assessment, risk of bias, menopausal status in breast cancer) explained the amount of between-study variance (*p* > 0.05).

The funnel plot for breast cancer as well as T2D did not suggest the presence of publication bias, and Egger’s test confirmed that there was no apparent evidence of bias. However, the funnel plot for IHD studies was asymmetrical, and the test for small-study effects evidenced that publication bias could be present (*p* < 0.001) (Supplementary file 6). Asymmetry was represented by the lack of small studies with null findings.

**Fig. 4:**
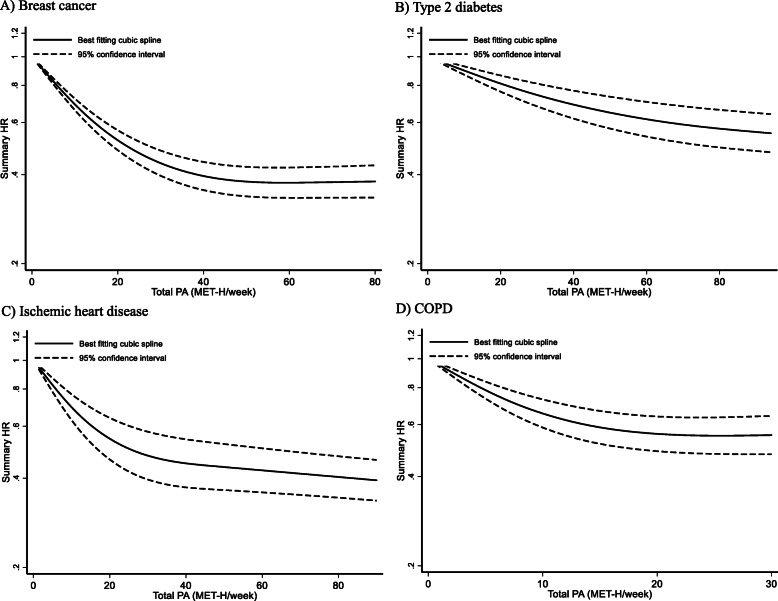
Non-linear dose–response meta-analysis for the association between post-diagnosis physical activity and all-cause mortality. **a**) breast cancer (*n* = 12); **b**) type 2 diabetes (*n* = 6); **c**) ischemic heart disease (*n* = 8); and **d**) COPD (*n* = 2). The figure includes values up to 100 MET-h/week

Figure 4 presents the non-linear dose–response meta-analysis among the four NCD populations. The results for breast cancer (*n* = 12), T2D (*n* = 6), IHD (*n* = 8) as well as COPD (*n* = 2) indicated a non-linear dose–response relationship between post-diagnosis physical activity presented in MET-h/week and all-cause mortality (*p*_for non-linearity_ < 0.001).

The curves for breast cancer, T2D and IHD show the steepest drop between 0 MET-h/week and 20 MET-h/week; the COPD curve drops more markedly between 0 and 10 MET-h/week. After this, the curves flatten out. For T2D and COPD, significantly higher physical activity levels (up to around 90 MET-h/week) are also associated with further positive effects on mortality rates. For breast cancer, there appears a plateau with no additional effects on mortality with more than 45 MET-h/week. For COPD, the curve could only be calculated up to 30 MET-h/week.

## Discussion

In this systematic review and meta-analysis, higher levels of post-diagnosis physical activity were associated with a reduction in all-cause mortality in adults with breast cancer, T2D, IHD and COPD. Our dose–response meta-analysis highlights a non-linear association between physical activity levels and mortality characterized by (1) no threshold for the beneficial effect of physical activity on mortality (i.e. even low levels of physical activity are beneficial for mortality rates compared to being physically inactive), (2) a non-linear curve, where the greatest difference in mortality rates occurs among inactive compared to minimally active individuals, and (3) for higher physical activity levels, the dose–response curves flatten out. Higher post-diagnosis physical activity levels may result in a slight reduction of all-cause mortality in breast cancer, T2D and IHD. The evidence is very uncertain about this effect in individuals with COPD.

The subgroup meta-analysis showed that longer follow-ups (≥10 years) lead to higher reductions of SHR. Although the effect size is higher for follow-ups that are 10 years or longer, there is an unexplained heterogeneity between the effects of physical activity within each subgroup. However, the unbalanced distribution of studies and the low overall number of studies for some subgroups make the interpretation difficult. Hence, it is uncertain whether the length of follow-up can explain the heterogeneity in the effect size. The reduction in mortality rates from physical activity were consistent and much the same after controlling for geographic areas (Asia, Europe, US, other), age (< 60 years; ≥60 years), number of cases (< 100, 100–500) and the risk of bias (moderate; serious). Due to a lack of studies, we were not able to determine dose–response relationships for physical activity and mortality in adults with low back pain, osteoarthritis, depressive disorder, lung cancer or stroke.

### Comparison with other studies

Our findings confirm previous reviews and linear meta-analyses, which showed a general correlation between higher physical activity levels and lower mortality rates in adults with T2D [6] and breast cancer [7, 55, 56]. For women with breast cancer, the one available randomized controlled trial reported HR for mortality from 0.45 (95% CI 0.21–0.97) for the physical activity intervention group compared to the control group [57]. However, since no specific activity levels are measured in this study, HRs are difficult to compare with the HR per each ten metabolic equivalent tasks hours calculated by us. Our linear meta-analysis reveals reductions of SHR per 10 MET-h/week that vary between the four NCDs. The lowest SHR reduction in our results was found in T2D (4%) – a somewhat weaker association than the 9.5% reduction per one MET-h/day reported by Kodama et al. [6]. Findings may differ because of the differences in a) the outcome measured (all-cause mortality vs. cause-specific mortality), b) in the physical activity categories (high vs. low vs dose-response analysis) and, c) in the included studies. We found medium reductions in IHD (12%) and breast cancer (22%) and the highest SHR reductions in COPD (30%). Our applied non-linear dose–response-analysis extends and refines these previous linear analyses. The associations between different post-diagnosis physical activity levels and mortality for adults with NCDs are very similar to those recently developed for the general population [9, 58, 59]. Therefore, our results confirm the following main characteristics of the dose–response curves in the general population for adults with selected NCDs: (1) no threshold for the positive effect, (2) the most pronounced SHR reductions occurs between adults with little physical activity compared to those being physically inactive, and (3) no negative effects on mortality at higher volumes of physical activity.

For higher volumes of physical activity equivalent to an energy expenditure of more than five times the weekly recommended moderate-intensity physical activity of 150 min and more, the dose–response curve is less clearly defined. The dose–response curves of the US Physical Activity Guidelines Advisory Committee [9] does not include physical activity levels of more than 30 MET-hours/week. Ekelund et al. [59] include higher volumes of physical activity, stating that the maximal reductions in SHR were seen at about 24 min/day of moderate to vigorous physical activity or 375 min/day of light intensity; higher volumes of physical activity are associated with a slight reduction in their benefit on mortality rates. In our study, higher physical activity levels were associated with continuously small declines in mortality rates for IHD and T2D. For breast cancer, there is a point of maximum reduction of SHR at 55 MET h/week with no additional benefits for higher physical activity levels. No data is available on higher physical activity levels for COPD.

In the total population, 70% of the maximum effect on mortality risk reduction is achieved at an energy consumption of 8.25 MET-h/week (equivalent to meeting the physical activity recommendations of 150 weekly minutes) [9]. Our results indicate that in adults with NCDs, this energy consumption is associated with about 40% of the maximum achievable reduction in mortality rates. Physical activity and both overall and cardiovascular mortality after stroke were connected through a dose–response relationship where 10 MET-h/day of physical activity produced 35–46% reductions in SHR [52]. Although one study reported data on stroke patients [52], it was not sufficient to be included in the meta-analysis. For 4 NCDs (low back pain, osteoarthritis, depressive disorder, lung cancer), we were not able to find appropriate studies for our analysis. Thus, our findings confirm the research gap in the clinical populations already identified before [9].

### Strengths and limitations

Our study has several strengths. Its main strength is the broad and comprehensive systematic literature search for 9 NCDs that have a high relevance for public health. For the first time, our work generates a broad overview of post-diagnosis physical activity and mortality for adults with NCDs. Another strength is the applied non-linear dose–response meta-analysis that enables precise statements regarding the effective dose of physical activity for reduced mortality rates. This information helps with the adaption or development of exercise recommendations for adults with NCDs. In addition, the use of the new ROBINS-I tool is a methodological strength that allows for a precise estimation of the risk of bias in different domains (e.g. bias in the measurement outcome, due to missing outcome data or due to deviation from intended interventions).

Despite its strengths, this systematic review and meta-analysis has limitations that should be acknowledged. First, at the outcome level of the study, the risk of bias in the measurement of physical activity in the original studies is unknown. All studies measured physical activity levels using self-reports. Compared to device-based measurements, self-reported measures are prone to over-reporting of one’s physical activity levels [60]. If the over-reporting of physical activity already plays a role at low physical activity levels, the actual high relative reduction in mortality rates of somewhat physically active persons compared to inactive persons could be underestimated. Most of the studies have measured the level of physical activity only at one point in time, thus meaning that no information on changes over time is available. Moreover, different cut-off points were used by the single studies to classify the participants’ levels of physical activity. This might lower the accuracy of the dose–response curves.

Second, the update of our literature search in August 2019 was conducted through Google Scholar and not through the original data bases (PubMed, Scopus and the Web of Science). This means that our original search strategy is not identical to the search update.

Third, at the study level, our findings are susceptible to bias derived from studies of an observational nature. Prospective observational cohort studies fail to provide conclusive evidence of a causal relationship between physical activity and mortality [61, 62]. Our results might be affected by reverse causality, as patients may tend to adjust their physical activity level according to the disease severity and prognosis. Consequently, our analysis of cohort studies does not provide a conclusive answer as to whether the reported dose–response relationships between physical activity and mortality are actually causal or only correlative. According to Hill [63], however, our results increase the sense of confidence in a causal relationship because they display (1) a clear dose–response curve, (2) a strong association or high effect size, and (3) consistent results in different studies. For the diseases with low certainty of evidence (breast cancer, IHD, T2D) the persistence of our findings for longer follow ups of > 10 years further strengthens our confidence in a causal relationship. Following the arguments from Hill [63], the additional proof of biological plausibility and evidence from experimental studies could further strengthen the confidence in causality. Bases on our results, we cannot exclude selection/survival bias, because inclusion of the participants in the studies depended on survival time after diagnosis of the disease. It is possible that participants with severe forms of the disease were already deceased or too ill to participate.

Fourth, at the review level and as reported in the study protocol [11], we did not consider the potential differences between different physical activity intensities (i.e. light vs moderate vs vigorous), between physical activity in different contexts (e.g. leisure time physical activity vs occupational physical activity) or the interaction between physical activity and sedentary behaviour. Furthermore, our analysis is likely to be affected by small-study effects and the small number of original studies available for the sensitivity analysis.

### Implications and future research

Assuming causality, our findings have implications for adults with NCDs, physicians and other health professionals involved in physical activity promotion and exercise therapy, as well as healthcare decisionmakers and policymakers. First, our results bear importance for policymakers and those involved in public health issues. The results highlight the importance of a physically active lifestyle and support strategies to promote physical activity (e.g. the World Health Organization’s Global Action Plan on Physical Activity) [64]. Second, for those creating physical activity guidelines, our findings may inform developments or updates on physical activity recommendations for adults with NCDs. Our findings reinforce low-dose physical activity recommendations that clearly demonstrate there is no minimum dose of physical activity and that effects on longevity occur at a volume of physical activity significantly below the recommended minimum dose of 150 min per week [65]. The “+10 minutes of physical activity per day” from Japan [66] or the “Every Step Counts” message from Germany [67] might be more feasible and efficient physical activity recommendations. Third, for physicians, our results illustrate the medical potential of exercise as medicine and encourage initiatives to anchor assess to and promotion of physical activity in routine medical care [68]. Fourth, for health professionals in the field of physical activity promotion, our results could lead to new targets for health-enhancing physical activity. Adults with NCDs are often rather physically inactive [69] and experience various barriers to physical activity, including time constraints and personal doubts about being able to participate in regular physical activity [70–72]. Completing at least 150 min of physical activity per week is considered by many to be overwhelming and unachievable. For adults with NCDs, non-threshold-based, low-dose physical activity recommendations could be effective while also being encouraging and easier to implement. Thus, low-dose physical activity recommendations would destroy many barriers in relation to an active lifestyle and increase the probability of success of interventions that promote physical activity.

The results also bear implications for future research. We identified a research gap: For 4 out of the 9 NCDs reviewed (lung cancer, depressive disorder, lung cancer, low back pain), there were no eligible studies available. Since the associations for post-diagnosis physical activity and mortality in adults with NCDs and the total population are different [8], future research should either conduct cohort studies on adults with NCDs or make a differentiation in the analysis of the total population between healthy people and those with an existing NCD. Furthermore, based on the considerable analyses of Ekelund et al. [59] in the overall population, future analyses for adults with NCDs should also consider different intensities and types of physical activity as well as the interaction between sedentary behaviour and physical activity. Finally, future studies should apply more reliable device-based assessments of physical activity instead of questionnaires that are prone to over-reporting.

## Conclusion

In conclusion, our systematic review and meta-analysis provides low certainty of evidence that higher levels of physical activity are associated with lower mortality rates in adults with T2D, IHD and breast cancer, while the certainty of evidence for COPD is very low. The shape of the dose-response curves are characterized by no threshold for the beneficial effect of physical activity on mortality meaning that any physical activity is better than none, and a regressive, non-linear dose-response pattern where the greatest difference in mortality rates occurs among inactive compared to minimally active individuals. There is no minimum dose of physical activity for life prolongation. Less physical activity than the recommended 150 min a week has life expectancy benefits for adults with a NCD. Our results encourage the development of low-dose physical activity recommendations for adults with NCDs.

## Supplementary information

**Additional file 1.** PRISMA Checklist.

**Additional file 2.** Search Results.

**Additional file 3.** Measurement of Physical Activity.

**Additional file 4.** Risk of Bias Summary

**Additional file 5.** GRADE Evidence Profiles.

**Additional file 6.** Funnel Plots.

**Additional file 7.** Subgroup Meta-Analysis.

## Data Availability

Data (including the extracted contents from the searched articles) are available upon reasonable request from Dr. Wolfgang Geidl; mail: wolfgang.geidl@fau.de

## Abbreviations

NCD: Noncommunicable disease
T2D: Type 2 diabetes
MET: Metabolic equivalent task
COPD: Chronic obstructive pulmonary disease
IHD: Ischemic heart disease
SHR: Standard hazard ratio
