# Supplementary material for: Dose–response relationship between physical activity and mortality in adults with noncommunicable diseases: a systematic review and meta-analysis of prospective observational studies
Source: Int J Behav Nutr Phys Act. 2020 Aug 26;17:109. doi: 10.1186/s12966-020-01007-5 (PMC7448980; doi:10.1186/s12966-020-01007-5)
Supplement: Supplementary file 2 — Additional file 2. Search Results. [file 12966_2020_1007_MOESM2_ESM.docx]

| **Supplementary file 2**. Search Results.  PubMed search results *(09.08.2018)* | |
| --- | --- |
| **Breast cancer** | |
| **#1** (("breast neoplasms"[MeSH Terms]) OR ("breast neoplasm"[Title/Abstract] OR "breast tumor"[Title/Abstract] OR "breast carcinoma"[Title/Abstract] OR "human mammary neoplasm"[Title/Abstract] OR "breast cancer"[Title/Abstract] OR "mammary cancer"[Title/Abstract] OR "breast malignant neoplasm"[Title/Abstract] OR "breast malignant tumor"[Title/Abstract])) | 342,536 |
| **#2** (("human activities" OR "motor activities" OR "leisure activities" OR "exercises" OR "running" OR "walking" OR "bicycling" OR "gardening" OR "sports" OR "activities of daily living"[MeSH Terms]) OR ("human activities"[Title/Abstract] OR "human activity"[Title/Abstract] OR "motor activity"[Title/Abstract] OR "motor activities"[Title/Abstract] OR "leisure activities"[Title/Abstract] OR "leisure activity"[Title/Abstract] OR "exercise"[Title/Abstract] OR "exercises"[Title/Abstract] OR "running"[Title/Abstract] OR "walking"[Title/Abstract] OR "bicycling"[Title/Abstract] OR "gardening"[Title/Abstract] OR "sports"[Title/Abstract] OR "sport"[Title/Abstract] OR "activities of daily living"[Title/Abstract] OR "physical activity"[Title/Abstract] OR "physical activities"[Title/Abstract] OR "nonexercise activity"[Title/Abstract] OR "nonexercise activities"[Title/Abstract] OR "energy expenditure"[Title/Abstract] OR "caloric expenditure"[Title/Abstract])) | 634,540 |
| **#3** (("mortality" [Title/Abstract] OR "death" [Title/Abstract] OR "survival" [Title/Abstract] OR "life expectancy" [Title/Abstract] OR "years of life lost"[Title/Abstract]) OR "mortality"[MeSH Terms])) | 1,963,937 |
| #1 AND #2 AND #3 | 865 |
| **Type 2 diabetes** | |
| **#4** ("diabetes mellitus, type 2"[MeSH Terms]) OR ("diabetes mellitus, type 2"[Title/Abstract] OR "non insulin dependent diabetes mellitus"[Title/Abstract] OR "ketosis resistant diabetes mellitus"[Title/Abstract] OR "stable diabetes mellitus"[Title/Abstract] OR "type 2 diabetes mellitus"[Title/Abstract] OR "NIDDM"[Title/Abstract] OR "maturity onset diabetes mellitus"[Title/Abstract] OR "MODY"[Title/Abstract] OR "slow onset diabetes mellitus"[Title/Abstract]) | 120,877 |
| #4 AND #2 AND #3 | 818 |
| **Chronic obstructive pulmonary disease** | |
| **#5** (("COPD" OR "pulmonary disease, chronic obstructive"[MeSH Terms])) OR ("COPD"[Title/Abstract] OR "chronic obstructive pulmonary disease"[Title/Abstract] OR "COAD"[Title/Abstract] OR "chronic obstructive airway disease"[Title/Abstract] OR "chronic obstructive lung disease"[Title/Abstract] OR "chronic airflow obstruction"[Title/Abstract]) | 78,701 |
| #5 AND #2 AND #3 | 1355 |
| **Ischemic heart diseases** | |
| **#6** (("myocardial ischemia" OR "coronary artery disease" OR "myocardial infarction"[MeSH Terms])) OR ("myocardial ischemia"[Title/Abstract] OR "coronary artery disease"[Title/Abstract] OR "myocardial infarction"[Title/Abstract]) | 356,364 |
| #6 AND #2 AND #3 | 5299 |
| **Major depressive disorder** | |
| **#7** ((depression[MeSH Terms]) OR depressive disorder, major[MeSH Terms]) OR depressive disorder[Title/Abstract]) OR depressive symptoms[Title/Abstract]) OR emotional depression[Title/Abstract]) | 215,873 |
| #7 AND #2 AND #3 | 950 |
| **Low back pain** | |
| **#8** ((low back pain[MeSH Terms]) OR lumbago[Title/Abstract]) OR low back ache[Title/Abstract]) | 20,634 |
| #8 AND #2 AND #3 | 25 |
| **Stroke** | |
| **#9** (("stroke"[MeSH Terms]) OR ("stroke"[Title/Abstract] OR "cerebrovascular accident"[Title/Abstract] OR "CVA"[Title/Abstract] OR "apoplexy"[Title/Abstract] OR "brain vascular accident"[Title/Abstract]) | 353,845 |
| 9# AND #2 AND #3 | 2415 |
| **Osteoarthritis** | |
| **#10** ((osteoarthritis[MeSH Terms]) OR osteoarthrosis[Title/Abstract]) OR osteoarthritides[Title/Abstract]) OR arthritis degenerative[Title/Abstract]) | 58,096 |
| #10 AND #2 AND #3 | 250 |
| **Lung cancer** | |
| **#11** ("lung neoplasms"[MeSH Terms]) OR ("lung neoplasm"[Title/Abstract] OR "pulmonary neoplasm"[Title/Abstract] OR "lung cancer"[Title/Abstract] OR "pulmonary cancer"[Title/Abstract]) | 254,893 |
| #11 AND #2 AND #3 | 536 |

| Scopus search results *(Assessed on 10.08.2018)* | |
| --- | --- |
| **Breast cancer** | |
| **#1** TITLE-ABS ("breast neoplasm" OR "breast tumor" OR "breast carcinoma" OR "human mammary neoplasm" OR "breast cancer" OR "mammary cancer" OR "breast malignant neoplasm" OR "breast malignant tumor" ) | 320,740 |
| **#2** TITLE-ABS ( "human activit*" OR "motor activit*" OR "physical activit*" OR "leisure activit*" OR "exercise" OR "running" OR "walking" OR "bicycling" OR "gardening" OR "sport*" OR "activit* of daily living" OR "nonexercise activit*" OR "energy expenditure" OR "caloric expenditure" ) | 1,091,721 |
| **#3** TITLE-ABS ( "mortality" OR "death" OR "survival" OR "life expectancy" OR "years of life lost" ) | 2,465,566 |
| #1 AND #2 AND #3 | 821 |
| **Type 2 diabetes** | |
| **#4** TITLE-ABS ( "diabetes mellitus, type 2" OR "non insulin dependent diabetes mellitus" OR "ketosis resistant diabetes mellitus" OR "stable diabetes mellitus" OR "type 2 diabetes mellitus" OR "NIDDM" OR "maturity onset diabetes mellitus" OR "MODY" OR "slow onset diabetes mellitus" ) | 57,592 |
| #4 AND #2 AND #3 | 495 |
| **Chronic obstructive pulmonary disease** | |
| **#5** TITLE-ABS ( "COPD" OR "chronic obstructive pulmonary disease" OR "COAD" OR "chronic obstructive airway disease" OR "chronic obstructive lung disease" OR "chronic airflow obstruction" ) | 70,715 |
| #5 AND #2 AND #3 | 1,291 |
| **Ischemic heart disease** | |
| **#6** TITLE-ABS ( "myocardial ischemia" OR "coronary artery disease" OR "myocardial infarction" ) | 286,103 |
| #6 AND #2 AND #3 | 5,373 |
| **Major depressive disorder** | |
| **#7** TITLE-ABS ( "depression" OR “depressive disorder "depressive disorder" OR "depressive symptoms" OR "emotional depression" ) | 454,118 |
| #7 AND #2 AND 3# | 2,269 |
| **Low back pain** | |
| **#8** TITLE-ABS ( "low back pain" OR "lumbago" OR "low backache" ) | 34,236 |
| #8 AND #2 AND 3# | 42 |
| **Stroke** | |
| **#9** TITLE-ABS ( "stroke" OR "cerebrovascular accident" OR "CVA" OR "apoplexy" OR "brain vascular accident" ) | 309,351 |
| #9 AND #2 AND 3# | 2,397 |
| **Osteoarthritis** | |
| **#10** TITLE-ABS ( "osteoarthritis" OR "osteoarthrosis" OR "osteoarthritides" OR "arthritis degenerative" ) | 68,550 |
| #10 AND #2 AND 3# | 271 |
| **Lung cancer** | |
| **#11** TITLE-ABS ( "lung neoplasm" OR "pulmonary neoplasm" OR "lung cancer" OR "pulmonary cancer" ) | 166,510 |
| #11 AND #2 AND 3# | 413 |

| Web of Science search results *(10.08.2018)* | |
| --- | --- |
| **Breast cancer** | |
| **#1** TOPIC: ("breast neoplasms" OR "breast neoplasm" OR "breast tumor” OR "breast carcinoma" OR "human mammary neoplasm” OR "breast cancer" OR "mammary cancer" OR "breast malignant neoplasm" OR "breast malignant tumor") OR TITLE: ("breast neoplasms" OR "breast neoplasm" OR "breast tumor” OR "breast carcinoma" OR "human mammary neoplasm” OR "breast cancer" OR "mammary cancer" OR "breast malignant neoplasm" OR "breast malignant tumor") | 463,023 |
| **#2** TOPIC: ("human activit*" OR "motor activit*" OR "physical activit*" OR "leisure activit*" OR "exercise" OR "running" OR "walking" OR "bicycling" OR "gardening" OR "sport*" OR "activit* of daily living" OR "nonexercise activit*" OR "energy expenditure" OR "caloric expenditure") OR TITLE: ("human activit*" OR "motor activit*" OR "physical activit*" OR "leisure activit*" OR "exercise" OR "running" OR "walking" OR "bicycling" OR "gardening" OR "sport*" OR "activit* of daily living" OR "nonexercise activit*" OR "energy expenditure" OR "caloric expenditure") | 649,212 |
| **#3** TOPIC: ("mortality" OR "death" OR "survival" OR "life expectancy" OR "years of life lost") OR TITLE: ("mortality" OR "death" OR "survival" OR "life expectancy" OR "years of life lost") | 2,049,912 |
| #1 AND #2 AND #3 | 1,617 |
| **Type 2 diabetes** | |
| **#4** TOPIC: ("diabetes mellitus, type 2" OR "diabetes mellitus, type 2" OR "non insulin dependent diabetes mellitus" OR "ketosis resistant diabetes mellitus" OR "stable diabetes mellitus" OR "type 2 diabetes mellitus" OR "NIDDM" OR "maturity onset diabetes mellitus" OR "MODY" OR "slow onset diabetes mellitus") OR TITLE: ("diabetes mellitus, type 2" OR "diabetes mellitus, type 2" OR "non insulin dependent diabetes mellitus" OR "ketosis resistant diabetes mellitus" OR "stable diabetes mellitus" OR "type 2 diabetes mellitus" OR "NIDDM" OR "maturity onset diabetes mellitus" OR "MODY" OR "slow onset diabetes mellitus") | 67,778 |
| #4 AND #2 AND #3 | 855 |
| **Chronic obstructive pulmonary disease** | |
| **#5** TOPIC: ("COPD" OR "pulmonary disease, chronic obstructive" OR "COPD" OR "chronic obstructive pulmonary disease" OR "COAD" OR "chronic obstructive airway disease" OR "chronic obstructive lung disease" OR "chronic airflow obstruction) OR TITLE: ("COPD" OR "pulmonary disease, chronic obstructive" OR "COPD" OR "chronic obstructive pulmonary disease" OR "COAD" OR "chronic obstructive airway disease" OR "chronic obstructive lung disease" OR "chronic airflow obstruction) | 66,350 |
| #5 AND #2 AND #3 | 1,476 |
| **Ischaemic heart diseases** | |
| **#6** TOPIC: ("myocardial ischemia" OR "coronary artery disease" OR "myocardial infarction" OR "myocardial ischemia" OR "coronary artery disease" OR "myocardial infarction") OR TITLE: ("myocardial ischemia" OR "coronary artery disease" OR "myocardial infarction" OR "myocardial ischemia" OR "coronary artery disease" OR "myocardial infarction") | 372,107 |
| #6 AND #2 AND #3 | 7,851 |
| **Major depressive disorder** |  |
| **#7** TOPIC: (“depression” OR “depressive disorder, major” OR “depressive disorder” OR “depressive symptoms” OR “emotional depression”) OR TITLE: (“depression” OR “depressive disorder, major” OR “depressive disorder” OR “depressive symptoms” OR “emotional depression”) | 351,657 |
| #7 AND #2 AND #3 | 2,933 |
| **Low back pain** | |
| **#8** TOPIC: (“low back pain” OR “lumbago” OR “low backache”) OR TITLE: (“low back pain” OR “lumbago” OR “low backache”) | 36,560 |
| #8 AND #2 AND #3 | 86 |
| **Stroke** | |
| **#9** TOPIC: ("stroke" OR "stroke" OR "cerebrovascular accident" OR "CVA" OR "apoplexy" OR "brain vascular accident") OR TITLE: ("stroke" OR "stroke" OR "cerebrovascular accident" OR "CVA" OR "apoplexy" OR "brain vascular accident") | 276,128 |
| #9 AND #2 AND #3 | 2,759 |
| **Osteoarthritis** | |
| #10 TOPIC: (“osteoarthritis“ OR “osteoarthrosis“ OR “osteoarthritides” OR “arthritis degenerative”) OR TITLE: (“osteoarthritis“ OR “osteoarthrosis“ OR “osteoarthritides” OR “arthritis degenerative”) | 75,978 |
| #10 AND #2 AND #3 | 361 |
| **Lung cancer** | |
| #11 TOPIC: ("lung neoplasms" OR "lung neoplasm" OR "pulmonary neoplasm" OR "lung cancer" OR "pulmonary cancer") OR TITLE: ("lung neoplasms" OR "lung neoplasm" OR "pulmonary neoplasm" OR "lung cancer" OR "pulmonary cancer") | 224,136 |
| #11 AND #2 AND #3 | 697 |

**Google Scholar Update** (05.08.2019)

| Breast cancer | 3569 |
| --- | --- |
| Type 2 diabetes | 1650 |
| Chronic obstructive pulmonary disease | 972 |
| Ischemic heart diseases | 731 |
| Stroke | 7 |
