# Supplementary material for: Dose–response relationship between physical activity and mortality in adults with noncommunicable diseases: a systematic review and meta-analysis of prospective observational studies
Source: Int J Behav Nutr Phys Act. 2020 Aug 26;17:109. doi: 10.1186/s12966-020-01007-5 (PMC7448980; doi:10.1186/s12966-020-01007-5)
Supplement: Supplementary file 3 — Additional file 3. Measurement of Physical Activity. [file 12966_2020_1007_MOESM3_ESM.docx]

**Supplementary file 3.** Measurement of physical activity across studies and main findings.

| **First author, year** | **Instrument** | **Domains covered: time post diagnosis** | **Psychometric properties** | **Main Findings** |
| --- | --- | --- | --- | --- |
| **Breast cancer** | | | |  |
| Ammitzbøll 2016 | EPIC physical activity questionnaire | Exercise (sports, walking, cycling), household activities (do-it-yourself, household, gardening), and total PA (exercise and household): measured after diagnosis, median (IQR), y = 3.5 (3) | Validated against an accelerometer[1]. Spearman rank correlation ρ = 0.29 and 0.21 for total PA and non-occupational PA respectively. Repeatability: weighted kappa = 0.62, 0.65 and 0.58 for total PA, non-occupational and recreational PA respectively. Validated against heart rate monitoring with individual calibration. Repeatability: weighted kappa = 0.6; P < 0.0001. Positive correlation between PA and resting metabolic rate (P < 0.003) and cardiorespiratory fitness (P < 0.001)[2]. | An existing relationship was found between exercise (≤ 2.5 h/week) and ACM (HR = 0.68; 95% CI: 0.47, 0.99), with a risk reduction of 44% between exercisers and non-exercisers (HR = 0.56; 95% CI: 0.33, 0.95). The association for household activities and total PA was not evident. Change in PA level from pre to post diagnosis was not shown to play a role in mortality prediction. No dose-response relationship was detected. |
| Bao 2015 | SWHS questionnaire | Exercise, up to 5 most common types: measured 6, 18, 36, 60 months post diagnosis | Validated by comparing with 7-day PA logs and 7-day physical activity questionnaire. Significant correlation was observed for PA log (first administration, r = 0.50; second administration, r = 0.74) and 7-day PAQ (first administration, r = 0.62; second administration, r = 0.80). Reproducibility of exercise participation (kappa = 0.64) and exercise energy expenditure (ICC = 0.70)[3]. | They found an inverse association between exercise measured 6, 18, 36, and 60 months after diagnosis and ACM as well as recurrence/BCSM. Exercisers (≥ 7.6 MET/h-week) have a 41% risk reduction for ACM and 43% for recurrence/BCSM when compared to non-exercisers, HR = 0.59 (95% CI: 0.38, 0.93) and HR = 0.57 (0.35, 0.92) respectively. Exercising at least 2.5 h/week produced a risk reduction of 40 % for any cause of death and 44% for recurrence/BCSM. |
| Bertram 2011 | Adapted WHI Personal Habits  Questionnaire | Walking, exercise: measured at baseline (up to 4 years post-diagnosis) and at 1-year follow-up. | Validated against accelerometry and Physical Activity Recall (PAR). WHI overestimated total PA by 6 minutes (P = 0.95), vigorous PA with 27 minutes (P < 0.001), and underestimated moderate PA with 21 minutes (P = 0.08). Correlation with the accelerometer and PAR were r = 0.73 (P < 0.01), and r = 0.88 (P < 0.001) respectively. ICC = 0.64 (0.47-0.78). Specificity = 0.60 and agreement kappa = 0.23[4]. Sensitivity for adherence to recommendations: 100%. | Inverse association was found between PA and mortality risk. Active women at baseline (≤ 24.7 MET-h/week) had a risk reduction of 53% compared with inactive women (HR = 0.47; 95% CI: 0.26, 0.84; p = .01). Those who were following aerobic PA recommendations (equivalent to > 10 MET-h/week) had a risk reduction of 35% (HR = 0.65, 95% CI: 0.47, 0.91; p < 0.01). Change in PA level from baseline to 1 year follow-up and ACM was significant only for women meeting guidelines at baseline with a risk reduction of 40%. |
| Bradshaw 2014 | Semi-open ended modified questionnaire (Bernstein 1994) | Recreational PA: measured yearly from 1 up to 5 years post-diagnosis. | No information about the validity of this instrument. | Post-diagnosis PA was inversely associated with survival. Highly active women (> 9 MET-h/week) in comparison with inactive ones (0 MET-h/week) had a risk reduction of 67% for ACM (HR = 0.33; 95% CI: 0.22, 0.48), and 73 % risk reduction for BCSM (HR = 0.27; 95% CI: 0.15, 0.46). This positive effect of PA was greater during two years after diagnosis. Women who were moderately active (0.1 – 9 MET-h/week) had lower but still important survival benefits HR = 0.43 (95% CI: 0.20, 0.83). |
| Chen 2011 | SWHS questionnaire | Exercise participation 6, 18, 36 months after diagnosis (5 most common activities) | Validated. See Bao et al. (2015). | Inverse relationship was found between exercise participation and ACM HR = 0.70 (95% CI: 0.56, 0 .88), and BCM HR = 0.60 (95% CI: 0.47, 0.76). Those expending less than 8.3 MET-h/week during 36 months after diagnosis had a risk reduction of 19% for ACM and 40% for BCSM. Women exercising 8.3 or more MET-h/week had better survival benefits for total mortality HR = 0.65 (95% CI: 0.51, 0.84). |
| de Glas 2014 | EPIC physical activity questionnaire | Recreational PA: walking, cycling, gardening, housekeeping, and sports: measured at 1 and 2 years post-diagnosis. | Validated against accelerometry. See above Ammitzbøll et al. 2016. | A significant relationship was present only for ACM. Women who expended 21.1 to 40 MET-h/week had the lowest risk for premature death HR = 0.43 (95% CI: 0.19, 0.94). Highly and very highly active women (40.1-65.5 MET-h/week) had around 40% risk reduction. |
| Holick 2008 | CWLS questionnaire | Recreational PA: walking outdoors; running (≥ 10 min/mile); lap swimming; tennis, squash, or racquetball; calisthenics, aerobics, or rowing machine; and other aerobic recreation (e.g., lawn mowing): measured at a median of 5.6 years after diagnosis. | Validated using past-week activity recalls and 7-day activity diaries[5].  Test-retest correlation coefficient for activity: 0.59  Test-retest for inactivity: 0.52  Correlation with past-week activity recall: 0.79  Correlation for inactivity: 0.44 | A clear dose-response relationship was observed for moderate intensity: for every 5 MET-h/week increase there was a risk reduction of 15% for BCSM (HR = 0.85, 95% CI: 0.74, 0.98; P for linear trend = 0.03). Vigorous intensity was not associated with BCSM. Using the lowest PA level as reference, overall and BCSM risk were 56% (HR=0.44, 95% CI: 0.32, 0.60) and 49 % (HR = 0.51, 95% CI: 0.29, 0.89) lower for those engaging in 21 or more MET-h/week. |
| Holmes 2005 | Questionnaire | Leisure-time PA: walking or hiking outdoors, jogging, running, bicycling, swimming, tennis, calisthenics, aerobics, aerobic dance, rowing machine, squash, racquet ball: measured biannually at least 2 years post-diagnosis, with a median of 38 months. | Validated again four 7-day activity diaries and 4 past-weeks questionnaires.  Total PA underestimated by 20%  Correlation coefficient r = 0.62 (95% CI: 0.44-0.75)  Intra-individual correlation for walking r = 0.70 (95% CI: 0.49-0.84) | PA higher than 3 MET-h/week was associated with significant decreased risk for both ACM and BCSM. The risk reduction for ACM varied from 29% for 3-8.9 MET-h/week to 44% for 15-23.9 MET-h/week, and for BCSM varied from 2% for 3-8.9 MET-h/week to 50% for 9-14.9 MET-h/week. P for trend < 0.05. This association was more pronounced in women with hormone-responsive tumors, where the most active women (> 9 MET-h/week) had a 50 % mortality risk reduction RR = 0.50 (95% CI: 0.34, 0.74). |
| Irwin 2008 | Modiﬁable Activity Questionnaire | Recreational, occupational, household activities: measured 6 ± 2 months (median 5 months), and 3 y (median 2.5) after diagnosis. | Validated against doubly labeled water (r = 0.73).  3 week test-retest reliability r = 0.92 | Active women at 2 years after diagnosis had a 67% risk reduction for ACM when compared with inactive ones (HR = 0.33, 95% CI: 0.15, 0.73). The risk reduction was lower for pre-diagnosis PA HR = 0.69 (95% CI: 0.45, 1.06). With regard to change of PA level pre to post-diagnosis, increase of PA was related to a risk reduction of 45% (HR = 0.55; 95% CI: 0.22, 1.38), while decrease of PA was related to greater risk (HR = 3.95; 95% CI: 1.45, 10.50). Moderate PA of any level was inversely associated with risk of dying earlier. |
| Irwin 2011 | Questionnaire | Sports/recreational PA: walking, aerobics, jogging, tennis, and swimming laps, biking outdoors, exercise machine, calisthenics, easy swimming, and popular or folk dancing: assessed 3 and 6 years post-diagnosis (mean time 1.8 ± 1.0 y). | Validated against accelerometry and 7-day PA recall. Validity: r = 0.73  Test-retest reliability: weighted κ = 0.53 to 0.72  Intraclass correlation = 0.77  100 % sensitivity for adherence to guidelines[4] | Adherence to PA recommendations after diagnosis with BC was associated with greater mortality risk reductions compared to inactivity. Women expending 9 or more MET-h/week had a lower risk of dying from ACM or BCSM, with HR 0.54 (0.38, 0.79) and 0.61 (0.35, 0.99) respectively (P < 0.05). Participation in moderate PA had approximately similar benefits in risk reduction for both ACM/BCSM. Change or maintenance of PA level at 9 or more MET-h/week from before to after diagnosis was also related to 33% risk reduction for ACM (HR = 0.67; 95% CI: 0.46, 0.96), and a slight reduction for BCSM. |
| Maliniak 2018 | Questionnaire | Recreational PA: walking, dancing, bicycling, aerobics, tennis or racquetball, jogging or running, lap swimming: measured 3.4 y (median) post-diagnosis. | No information. | A significant inverse relationship was observed between pre- and post-diagnosis PA and overall mortality.  No association was observed between pre- and post-diagnosis PA and BCSM in women aged ≥ 65 years old. Women aged 46-64 years and expending 8.75 - < 17.5 MET-h/week had a risk reduction of 33% for ACM (HR = 0.67; 95% CI: 0.44 - 1.02; P-trend = 0.04) and 26% for BCSM (HR = 0.74; 95% CI: 0.38 - 1.43; P-trend = 0.01). More participation in LTPA was associated with 51% risk reduction. Women aged 65-92 years had 19% to 26% reduced risk of dying from any cause of death (P-trend < 0.001). |
| Sternfeld 2009 | Arizona Activity Frequency Questionnaire | Work-related, no work-related activities, household and caregiving, recreation, transportation: assessed 1.9 ± 0.6 y after diagnosis. | Validated against doubly labeled water[6].  Underestimation of PA energy expenditure: 13%  Correlation for total energy expenditure: r = 0.70 (P < 0.001) | Inverse relationship between moderate intensity PA and ACM was observed (HR = 0.66; 95%CI: 0.42–1.03; P-trend = 0.04). The association between PA expressed in MET-h/week or hours per week and breast cancer recurrence and death was not significant after adjusting for confounders. No association was observed for vigorous intensity PA. Time spent on moderate intensity PA showed to have a significant effect on longevity. |
| **Type 2 diabetes** | | | |  |
| Glenn 2015 | SCCS physical activity questionnaire | Household, occupation, leisure PA: measured at baseline. | Validated against the RT3 accelerometer and a last month PA survey[7].  Fair to moderate test-retest reliability: r = 0.22 to 0.47  Criterion validity: r = 0.17 to 0.31 | Dose-response trend was observed, where adults expending 24.9 or more MET-h/day (equivalent to 1 hour of moderate exercise five times per week) had a 36 % mortality risk reduction (HR = 0.64; 95% CI: 0.57–0.73; P-trend <.0001). The increase in sedentary time increased vulnerability to premature death (HR = 1.21; 95% CI: 1.08–1.37). The combined effect of active and sedentary behavior, a positive relationship between PA and risk of death was observed across all levels of sedentary time. |
| Gregg 2003 | National Health Interview physical activity survey | Walking; sports: gardening, jogging, aerobics, cycling, swimming, weight lifting, golf, basketball tennis: measured at baseline (mean time since diagnosis, 11 y). | Not validated. | A significant inverse relationship between walking, total PA and ACM was observed. Compared to non-walkers, those who walked or participated in recreational PA for 2 or more hours per week had 39 % (HR = 0.61; 95% CI: 0.48, 0.78; P-trend < 0.001) and 29 % (HR = 0.71; 95% CI: 0.59, 0.87; P-trend = 0.003) risk reduction of dying from any cause. The risk reduction for CVD mortality was 34 % (P-trend = 0.08) and 24 % (P-trend = 0.19) respectively. The lowest death risk reduction was observed in adults walking 3 to 3.9 hours per week (HR = 0.46; 95% CI: 0.29-0.71; P-trend = 0.004). With regard to intensity, those who walked in a self-perceived moderate pace had the grater benefit (HR = 0.57; 95% CI: 0.41-0.80; P-trend = 0.006). |
| Hu 2004 | Questionnaire | Occupational, commuting, recreational PA: measured at baseline. | Questions were reported to be similar to the questions used in the Seven Countries Study, which were validated [8, 9]. However, authors reported that the questionnaire itself was not validated. | There was a significant inverse association between all intensity levels of LTPA and both ACM and CVD mortality. Compared to light intensity PA, those engaging in moderate PA had a mortality risk reduction of approximately 17 % (P-trend < 0.05). For high intensity activities the risk of ACM or CVD was even lower, ranging from 29% to 33% (P-trend < 0.05). Similar relations were observed for occupational and commuting PA, though the later did not remain significant after adjusting for the two other types of PA. |
| Sluik 2012 | EPIC physical activity questionnaire; Cambridge Physical Activity Index | Total PA (occupational PA, cycling, sports); Leisure-time PA (walking, cycling, gardening, sports, household work, do-it-yourself activities); Walking: measured at baseline. | Validated against accelerometry[1]. See above Ammitzøll et al. (2016). | An inverse association was observed between total PA, LTPA, walking, and mortality outcomes. This relationship was slightly J shaped for total PA. When compared with the inactive group, HR for active, moderately active and moderately inactive group were 0.74 (95% CI: 0.59, 0.94), 0.62 (95% CI: 0.49, 0.78), and 0.69 (95% CI: 0.57, 0.83) respectively (P for trend = 0.001). HR for CVD mortality were 0.62 (95% CI: 0.38, 1.01), 0.51 (95%: 0.32, 0.81), and 0.65 (95% CI: 0.46, 0.91) respectively. |
| Sone 2013 | Questionnaire | Leisure-time PA: walking, jogging, cycling, golf, tennis, swimming, aerobics dancing, other exercise: measured at baseline. | The questionnaire resembled to the validated questionnaire of the Health Professionals’ Follow-up Study. | Diabetic patients participating in ≥ 15.4 MET-h/week of leisure-time PA had a significant lower risk of dying earlier than those expending 3.7 or less MET-h/week (HR = 0.47; 95% CI: 0.22, 0.99, p-trend = 0,046). Those expending 3.8 - 15.3 MET-h/week had a 12% risk reduction for dying of any cause (HR = 0.88, 95% CI: 0.47, 1.64). |
| Tanasescu 2003 | Questionnaire | Leisure-time PA: walking, hiking, jogging, running, bicycling, lap swimming, tennis, squash, racquetball, calisthenics, rowing, heavy outdoor work, weight lifting: measured biennially from 1986 to 1998. | Validated against a 1-week activity dairy administered in 4 different times during one year. Correlation for total PA: 0.65, vigorous PA: 0.58, and non-vigorous PA: 0.28. | Data revealed an inverse relationship between PA and ACM. Expending 37.2 or more MET-h/week was associated with a risk reduction of 42% compared to the most inactive group (RR = 0.58; 95%CI: 0.41, 0.83; p-trend = 0.005). Same trend was observed for individuals walking 16.1 MET-h/week or more, with a risk reduction of 43% (p-trend = 0.002). Walking pace played also a protective role against mortality despite the duration. |
| **Chronic obstructive pulmonary disease** | | | |  |
| Cheng 2018 | Physical Activity and Sedentary Behavior Assessment Questionnaire | Domestic PA, manual work, gardening, do-  it-yourself activities, walking, sports and exercise: measured at baseline. | Validated versus accelerometry[10].  Criterion validity: Spearman's correlation coefficient (ρ) for total activity = 0.30 in women, ρ = 0.20 in men.  ρ for MVPA = 0.42 for women, ρ = 0.38 for men | A linear dose-response relationship was noticed between total, MVPA, LTPA and overall and type-specific mortality. In contrast, domestic PA was not related to mortality. Meeting the PA recommendations (≥7.5 MET-h/week) was associated with significant risk reductions, specifically 44% for ACM (HR = 0.56, 95% CI: 0.45, 0.69), 52 % for CVD-specific death (HR = 0.48, 95% CI: 0.32 – 0.71), and 60% for respiratory mortality (HR = 0.40, 95%: 0.24, 0.67). Similar associations were present for MVPA, walking, sports and exercise |
| Garcia-Aymerich 2006 | Questionnaire by Saltin and Grimby | Occupational PA, leisure-time PA, jogging, cycling in summer, cycling in winter: measured at baseline. | Reported from the authors to have restricted validity. See below Lahtinen et al. (2017). | Higher levels of PA (low, moderate, high) were associated with lower hospital admissions and mortality hazard when compared to very low PA levels. The respective multi-variable adjusted HR for overall, CVD and respiratory mortality were 0.76 (95% CI: 0.65, 0.90), 0.77 (95% CI: 0.58, 1.02), and 0.70 (95% CI: 048, 1.02). The prognostic effect of PA in COPD patients was observed even for small activity doses. |
| **Ischemic heart diseases** | | | |  |
| Gerber 2011 | Questionnaire | Leisure-time PA: measured at 3-6 months, and 1–2, 5, 10–13 years after MI. | No information. | LTPA before MI did not play a role on mortality. On contrary, PA after MI was strongly associated with mortality in a dose-response gradient. The HRs for the regularly and irregularly active group compared to the inactive individuals were 0.71 and 0.56 respectively. |
| Janssen 2006 | Interview | Leisure-time PA: walking, swimming, hiking, aerobics, tennis, jogging, racquetball, golfing, bicycling, dancing, calisthenics, exercise bicycle, and two other sports indicated from the patient: assessed at baseline and at 3 years follow-up (N = 785). | No information. | The analysis revealed a consistent dose-response curvilinear relationship between PA and mortality risk. The multivariable adjusted hazard ratios for a weekly energy expenditure of < 500 kcal, 500 to 999, 1000 – 1999, 2000 – 2999 and ≥ 3000 kcal were 0.87, 0.77, 0.54 and 0.63 respectively (P-trend < 0.001). Reduced levels of PA at 3 years follow-up were also associated with increased risk, where a decrease of 1000 kcal/week was related to 24% increased mortality risk. Intensity levels of PA played no role on mortality. |
| Lahtinen 2017 | Saltin-Grimby Physical Activity Level Scale | Leisure-time PA: measured at baseline and 2 years follow-up. | Good validity. A study from Aires et al. (2003) confirmed the validity of self-reported leisure-time PA over a period of 25 years[11]. | LTPA was inversely associated with overall and cardiac mortality. Change in LTPA level from baseline to 2 years, despite the amount of change, was associated with reduced mortality risk and was considered to have prognostic significance. Becoming/ remaining inactive increased the risk of cardiac death by 2.4 and 4.9 fold in comparison to patients who remained or became active during the follow-up. |
| Moholdt 2017 | Questionnaire | Leisure-time PA: measured at least in one of three HUNT waves: H1, H2, H3. | The H1 and H3 questionnaires were similar[12]. Questions have been validated against VO_2_peak, the activity monitor ActiReg and the International Physical Activity Questionnaire[13]. | Patients following or exceeding PA recommendations had a lower risk of both ACM and CVD mortality, independent of BMI. Compared with inactive patients, the multivariable adjusted HRs for those in the low, recommended and high group were 0.85, 0.81, and 0.82 for ACM, and 0.87, 0.80 and 0.78 for CVD mortality. Patients who engaged in PA more than the recommended level did not have additional survival benefits. |
| Mons 2014 | Questionnaire | Leisure-time PA (strenuous): measured 1, 3, 6, 8 and 10 years after rehabilitation. | No information | The relationship between PA and mortality had a reverse J-shape form. Inactive patients had the highest risk of mortality in comparison with those engaging in LTPA 2 to 4 times per week. The adjusted HRs using time-dependent covariates were 3.81 (95% CI: 2.17, 6.70) for ACM and 3.39 (95% CI: 1.62, 7.10) for CVD mortality. In contrast, the group with a daily participation in PA had an increased risk of dying from ACM (HR = 1.77; 95% CI: 0.90, 3.47) or from CVD (HR = 2.37; 95% CI: 1.05, 5.34) when compared to the reference group. |
| Stewart 2017 | International Physical Activity Questionnaire | Total PA: habitual exercise, leisure-time and occupational PA: assessed at baseline (3.42 ± 6.38 years from diagnosis to randomization) | Validated against CSA accelerometer, and showed acceptable psychometric properties[14]. Criterion validity: rho = 0.30. | There was a non-linear relationship between habitual exercise and mortality outcomes, where the sedentary subjects can have a greater survival benefit from mild to moderate intensity PA, and active subjects have a less pronounced risk reduction. After adjusting for covariates, increase of the exercise volume was associated with a risk reduction of 10% ACM (HR = 0.90, 95% CI: 0.87, 0.93), and 8% for CVD mortality (HR = 0.92; 95% CI: 0.88, 0.96). Same, a two-fold increase of the exercise intensity was associated with a reduced mortality risk of 16% from ACM to 19% from CVD. Increase of exercise duration had also survival benefits. |
| Tian 2017 | Questionnaire | Occupational, commuting, household and leisure-time PA: measured at baseline. | The questionnaire combined questions from the EPIC-Norfolk [15] and the Shanghai Women's Health Study questionnaires[3, 16]. The final modified PA questionnaire was not directly validated[16]. | An inverse dose-response relationship was observed. Per 10 MET increase in daily PA the survival benefits for subjects with preexisting IHD were 38% (HR = 0.62; 95% CI: 0.54, 0.71) for ACM and 34% for CVD mortality (HR = 0.66, 95% CI: 0.55, 0.80). Improving daily health habits such as additional 60 gram of fresh fruits and 11 MET-h of PA, significantly reduced the mortality risk by approximately 40%. For individuals diagnosed with IHD at baseline, the multivariable adjusted HR for ACM and the 2^nd^ and 3^rd^ tertile of daily PA were 0.80 and 0.59 respectively. Similarly, the risk of dying from CVD was reduced by 22% to 34%. |
| Wannamethee 2000 | Questionnaire | Leisure-time PA (gardening, pleasure walking, do-it-yourself activities), sport (running, golf, swimming, tennis, sailing, digging), regular walking or cycling: measured at baseline and 12 to 14 years follow-up | The physical index used to classify participant according to the total score was validated. | When compared to the inactive or occasional active group, the fully adjusted RR for light, moderate and MVPA and ACM were 0.42, 0.47 and 0.63 respectively. The data showed that PA of light to moderate intensity is more beneficial for older man diagnosed with IHD. Similar benefits were observed for death from CVD. LTPA more than 4 hours per weekend and walking for > 40 minutes per day were associated with 59% and 52% lower risk reduction respectively. Moderate or heavy gardening and participation in one or more sport activities monthly had also survival benefits. |

SWHS, Shanghai Women’s Health Study; WHI, Women's Health Initiative, EPIC, European Prospective Investigation Into Cancer, CWLS, Collaborative Women’s Longevity Study; IQR, Interquartile range; PA, physical activity; MVPA, moderate-to-vigorous physical activity; IPAQ, International Physical Activity Questionnaire; ACM, all-cause mortality; BCM, breast cancer-specific mortality; HR, hazard ratio;

**References**

1 Cust AE, Smith BJ, Chau J, et al. Validity and repeatability of the EPIC physical activity questionnaire: a validation study using accelerometers as an objective measure. *Int J Behav Nutr Phys Act* 2008;5:33.

2 Wareham NJ, Jakes RW, Rennie KL, et al. Validity and repeatability of a simple index derived from the short physical activity questionnaire used in the European Prospective Investigation into Cancer and Nutrition (EPIC) study. *Public Health Nutr* 2003;6(4):407–13.

3 Matthews CE, Shu X-O, Yang G, et al. Reproducibility and validity of the Shanghai Women's Health Study physical activity questionnaire. *Am J Epidemiol* 2003;158(11):1114–22.

4 Johnson-Kozlow M, Rock CL, Gilpin EA, et al. Validation of the WHI brief physical activity questionnaire among women diagnosed with breast cancer. *Am J Health Behav* 2007;31(2):193–202.

5 Wolf AM, Hunter DJ, Colditz GA, et al. Reproducibility and validity of a self-administered physical activity questionnaire. *Int J Epidemiol* 1994;23(5):991–99.

6 Staten LK, Taren DL, Howell WH, et al. Validation of the Arizona Activity Frequency Questionnaire using doubly labeled water. *Med Sci Sports Exerc* 2001;33(11):1959–67.

7 Buchowski MS, Matthews CE, Cohen SS, et al. Evaluation of a Questionnaire to Assess Sedentary and Active Behaviors in the Southern Community Cohort Study. *J Phys Act Health* 2011;9(6):765–75.

8 Keys A, Aravnis C. Seven countries: A multivariate analysis of death and coronary heart disease. Cambridge: Harvard University Press 1980.

9 Sallis JF, Haskell WL, Wood PD, et al. Physical activity assessment methodology in the Five-City Project. *Am J Epidemiol* 1985;121(1):91–106.

10 Scholes S, Coombs N, Pedisic Z, et al. Age- and sex-specific criterion validity of the health survey for England Physical Activity and Sedentary Behavior Assessment Questionnaire as compared with accelerometry. *Am J Epidemiol* 2014;179(12):1493–502.

11 Aires N, Selmer R, Thelle D. The validity of self-reported leisure time physical activity, and its relationship to serum cholesterol, blood pressure and body mass index. A population based study of 332,182 men and women aged 40-42 years. *Eur J Epidemiol* 2003;18(6):479–85.

12 Moholdt T, Wisløff U, Lydersen S, et al. Current physical activity guidelines for health are insufficient to mitigate long-term weight gain: More data in the fitness versus fatness debate (The HUNT study, Norway). *Br J Sports Med* 2014;48:1489–96.

13 Kurtze N, Rangul V, Hustvedt B-E, et al. Reliability and validity of self-reported physical activity in the Nord-Trøndelag Health Study: HUNT 1. *Scand J Public Health* 2008;36(1):52–61.

14 Craig CL, Marshall AL, Sjostrom M, et al. International physical activity questionnaire: 12-country reliability and validity. *Med Sci Sports Exerc* 2003;35(8):1381–95.

15 Wareham NJ, Jakes RW, Rennie KL, et al. Validity and repeatability of the EPIC-Norfolk Physical Activity Questionnaire. *Int J Epidemiol* 2002;31(1):168–74.

16 Du H, Bennett D, Li L, et al. Physical activity and sedentary leisure time and their associations with BMI, waist circumference, and percentage body fat in 0.5 million adults: the China Kadoorie Biobank study. *The American journal of clinical nutrition* 2013;97(3):487–96.
