# Supplementary material for: Dose–response relationship between physical activity and mortality in adults with noncommunicable diseases: a systematic review and meta-analysis of prospective observational studies
Source: Int J Behav Nutr Phys Act. 2020 Aug 26;17:109. doi: 10.1186/s12966-020-01007-5 (PMC7448980; doi:10.1186/s12966-020-01007-5)
Supplement: Supplementary file 4 — Additional file 4. Risk of Bias Summary [file 12966_2020_1007_MOESM4_ESM.pdf]

Supplementary file 4. Risk of bias summary.

	Confounding	Selection of participants	Classification of physical activity	Departures from the intended interventions	Missing data	Measurement of mortality	Selective reporting	Overall bias
Ammitzbøll 2016	− (Serious)	+ (Moderate)	? (No information)	− (Serious)	− (Serious)	+ (Low)	+ (Low)	− (Serious)
Bao 2015	+ (Moderate)	+ (Low)	? (No information)	+ (Moderate)	+ (Low)	+ (Low)	+ (Low)	+ (Moderate)
Bertram 2011	+ (Moderate)	+ (Low)	? (No information)	+ (Low)	+ (Moderate)	+ (Low)	+ (Low)	+ (Moderate)
Bradshaw 2014	+ (Moderate)	+ (Low)	− (Serious)	+ (Moderate)	− (Serious)	+ (Low)	+ (Low)	− (Serious)
Chen 2011	+ (Moderate)	+ (Low)	? (No information)	+ (Low)	+ (Moderate)	+ (Low)	+ (Low)	+ (Moderate)
de Glas 2014	+ (Moderate)	+ (Low)	? (No information)	+ (Low)	+ (Moderate)	? (No information)	+ (Low)	+ (Moderate)
Holick 2008	+ (Moderate)	− (Serious)	? (No information)	− (Serious)	+ (Moderate)	+ (Low)	+ (Low)	− (Serious)
Holmes 2005	− (Serious)	+ (Moderate)	? (No information)	− (Serious)	+ (Moderate)	+ (Low)	+ (Low)	− (Serious)
Irwin 2008	− (Serious)	+ (Moderate)	? (No information)	− (Serious)	+ (Moderate)	+ (Low)	+ (Low)	− (Serious)
Irwin 2011	+ (Moderate)	+ (Low)	? (No information)	+ (Moderate)	+ (Moderate)	+ (Low)	+ (Low)	+ (Moderate)
Maliniak 2018	+ (Moderate)	+ (Low)	? (No information)	− (Serious)	+ (Moderate)	+ (Low)	+ (Low)	− (Serious)
Sternfeld 2009	− (Serious)	− (Serious)	? (No information)	− (Serious)	− (Serious)	+ (Low)	+ (Low)	− (Serious)
Glenn 2015	− (Serious)	+ (Low)	? (No information)	− (Serious)	+ (Low)	+ (Low)	+ (Low)	− (Serious)
Gregg 2003	− (Serious)	+ (Low)	− (Serious)	− (Serious)	+ (Moderate)	+ (Low)	+ (Low)	− (Serious)
Hu 2004	− (Serious)	+ (Moderate)	− (Serious)	− (Serious)	+ (Low)	+ (Low)	+ (Low)	− (Serious)
Sluik 2012	− (Serious)	+ (Low)	? (No information)	− (Serious)	+ (Low)	+ (Low)	+ (Low)	− (Serious)
Sone 2013	− (Serious)	+ (Moderate)	? (No information)	− (Serious)	+ (Low)	+ (Low)	+ (Low)	− (Serious)
Tanasescu 2003	+ (Moderate)	+ (Moderate)	? (No information)	+ (Moderate)	? (No information)	+ (Low)	+ (Low)	+ (Moderate)
Cheng 2018	− (Serious)	+ (Low)	? (No information)	− (Serious)	+ (Moderate)	+ (Low)	+ (Low)	− (Serious)
Garcia-Aymerich 2006	− (Serious)	+ (Low)	? (No information)	− (Serious)	+ (Low)	+ (Low)	+ (Low)	− (Serious)
Gerber 2011	+ (Moderate)	+ (Low)	? (No information)	+ (Moderate)	+ (Low)	+ (Low)	+ (Low)	+ (Moderate)
Janssen 2006	+ (Moderate)	+ (Low)	? (No information)	+ (Moderate)	+ (Moderate)	+ (Low)	+ (Low)	+ (Moderate)
Lahtinen 2018	+ (Moderate)	+ (Low)	? (No information)	+ (Moderate)	+ (Moderate)	+ (Low)	+ (Low)	+ (Moderate)
Moholdt 2017	+ (Moderate)	+ (Low)	? (No information)	+ (Moderate)	? (No information)	+ (Low)	+ (Low)	+ (Moderate)
Mons 2014	− (Serious)	+ (Low)	− (Serious)	+ (Moderate)	? (No information)	+ (Low)	+ (Low)	− (Serious)
Stewart 2017	− (Serious)	+ (Low)	? (No information)	− (Serious)	+ (Low)	? (No information)	+ (Low)	− (Serious)
Tian 2017	− (Serious)	− (Serious)	− (Serious)	− (Serious)	+ (Low)	+ (Low)	+ (Low)	− (Serious)
Wannamethee 2000	+ (Moderate)	+ (Moderate)	? (No information)	+ (Moderate)	+ (Low)	+ (Low)	+ (Low)	+ (Moderate)

+ Low: comparable to a well-performed RCT; + Moderate: sound evidence but not comparable to a well-performed RCT; − Serious: the study has some important problems; − Critical: the study is to critical to provide any useful evidence and should not be included in any synthesis; ? No information: not enough information to reach a judgment.
