# Supplementary material for: Dose–response relationship between physical activity and mortality in adults with noncommunicable diseases: a systematic review and meta-analysis of prospective observational studies
Source: Int J Behav Nutr Phys Act. 2020 Aug 26;17:109. doi: 10.1186/s12966-020-01007-5 (PMC7448980; doi:10.1186/s12966-020-01007-5)
Supplement: Supplementary file 5 — Additional file 5. GRADE Evidence Profiles. [file 12966_2020_1007_MOESM5_ESM.docx]

Supplementary File 5. GRADE evidence profiles.

| **Certainty assessment** | | | | | | | | **№ of participants** | | **Effect** | **Certainty** |
| --- | --- | --- | --- | --- | --- | --- | --- | --- | --- | --- | --- |
| **№ of studies** | **Study design** | **Risk of bias** | **Inconsistency** | **Indirectness** | **Imprecision** | **Publicaction bias** | **Other considerations** | **Participants** | **Cases** | **Summary relative effect (95% CI)** |  |
| **All-cause mortality for breast cancer (assessed with: HR)** | | | | | | | | | | | |
| 12 | observational studies | serious^a^ | not serious^b^ | Serious^c^ | not serious | undetected | dose‑response^d^gradient | 27.248 | 2867 | 0.78  (0.71 to 0.86) | ⨁⨁◯◯ LOW |
| **All-cause mortality for COPD (assessed with: HR)** | | | | | | | | | | | |
| 2 | observational studies | Serious^e^ | not serious^b^ | Serious^c^ | Serious^f^ | strongly suspected^f^ | dose‑response gradient^d^ | 4.784 | 1996 | 0.70  (0.45 to 1.09) | ⨁◯◯◯ VERY LOW |
| **All-cause mortality for IHD (assessed with: HR)** | | | | | | | | | | | |
| 8 | observational studies | Serious^h^ | not serious^b^ | Serious^c^ | not serious | undetected | dose‑response gradient^d^ | 42.027 | 7666 | 0.88  (0.83 to 0.93) | ⨁⨁◯◯ LOW |
| **All-cause mortality for type 2 diabetes (assessed with: HR)** | | | | | | | | | | | |
| 6 | observational studies | Serious^a^ | not serious^b^ | serious^b^ | not serious | undetected | dose-response gradient^d^ | 32.221 | 5630 | 0.96  (0.93 to 0.99) | ⨁⨁◯◯ LOW |

Explanations. CI: Confidence interval; a. Downgraded by two levels since five studies were judged as serious risk of bias regarding confounding or selection bias based on ROBINS-I; b. Despite the high I^2^ judged as not serious because of the overlapping CI and same direction of effects in the forest plots; c. Downgraded by one level because although exposure was assessed in all studies using validated questionnaires, there were differences in the assessment and calculation of physical activity levels; d. Upgraded by one level due to the dose-response gradient; e. Downgraded by two levels since two studies were judged as serious risk of bias regarding confounding or selection bias based on ROBINS-I; f. Downgraded by one level because the 95% CI includes the null value (HR=1,0) and includes important benefits HR<0.75; g. Downgraded by one level because publication bias could not be assessed due to limited number of studies (< 5 studies); h. Downgraded by two levels since two three studies were judged as serious risk of bias regarding confounding or selection bias based on ROBINS-I.
