# Supplementary material for: Dose–response relationship between physical activity and mortality in adults with noncommunicable diseases: a systematic review and meta-analysis of prospective observational studies
Source: Int J Behav Nutr Phys Act. 2020 Aug 26;17:109. doi: 10.1186/s12966-020-01007-5 (PMC7448980; doi:10.1186/s12966-020-01007-5)
Supplement: Supplementary file 6 — Additional file 6. Funnel Plots. [file 12966_2020_1007_MOESM6_ESM.docx]

**Supplementary file 6.** Funnel plots with pseudo 95% confidence limits.

a) Breast cancer b) Ischemic heart disease

****Egger’s test for small study effects: 0.787 Egger’s test for small study effects: <0.001


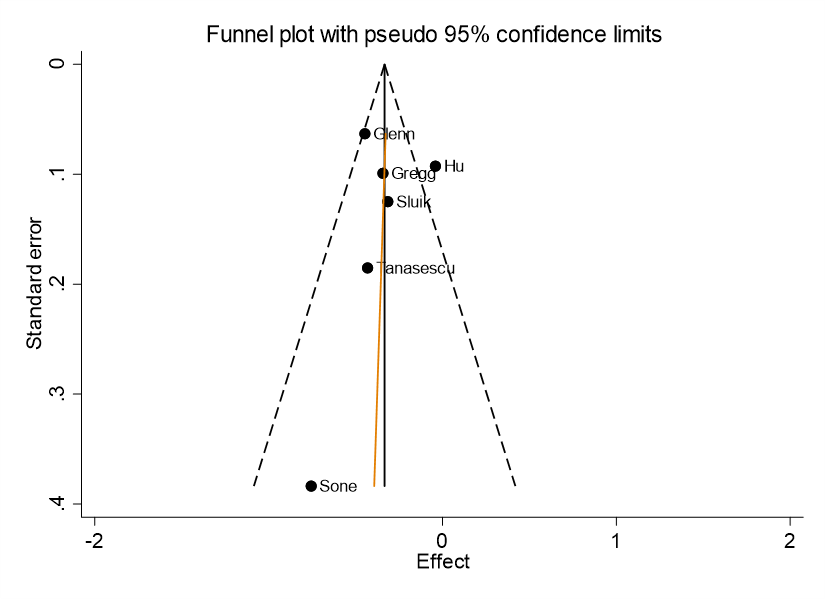
c) Type 2 diabetes

Egger’s test for small study effects: 0.826
