# Supplementary material for: Dose–response relationship between physical activity and mortality in adults with noncommunicable diseases: a systematic review and meta-analysis of prospective observational studies
Source: Int J Behav Nutr Phys Act. 2020 Aug 26;17:109. doi: 10.1186/s12966-020-01007-5 (PMC7448980; doi:10.1186/s12966-020-01007-5)
Supplement: Supplementary file 7 — Additional file 7. Subgroup Meta-Analysis. [file 12966_2020_1007_MOESM7_ESM.docx]

**Supplementary file 7.** Summary Hazard Ratios (SHR) and 95% CI from subgroup meta-analysis for physical activity and mortality (linear dose-response analysis, per 10 MET-hours/week).

**Table S7.1.** Breast cancer.

|  | | | **N of studies** | **SHRs**  **(95% CI)** * | ***Tau^2^*** | ***I^2^* (%)** | ***P*_within_ _subgroup_** † | ***P*_between_ _subgroup_** ‡ |
| --- | --- | --- | --- | --- | --- | --- | --- | --- |
| All studies | | | 12 | 0.78 (0.71, 0.86) | 0.020 | 90.1 | <0.001 | - |
| Menopausal status^#^ | | |  |  |  |  |  | 0.953 |
|  | | pre-and postmenopausal | 8 | 0.73 (0.64; 0.84) | 0.028 | 86.1 | <0.001 |  |
|  | | postmenopausal | 3 | 0.79 (0.61; 1.01) | 0.043 | 96.6 | <0.001 |  |
| Assessment of physical activity | | |  |  |  |  |  | 0.523 |
|  | | questionnaire | 10 | 0.84 (0.77, 0.91) | 0.013 | 87.5 | <0.001 |  |
|  | | interview | 2 | 0.54 (0.36, 0.81) | 0.067 | 80.1 | <0.001 |  |
| Geographic area | | |  |  |  |  |  | 0.658 |
|  | Asia | | 2 | 0.64 (0.52, 0.79) | 0.0 | 0.0 | 0.791 |  |
|  | Europe | | 1 | 0.99 (0.96, 1.02) | 0.0 | - | - |  |
|  | USA | | 8 | 0.74 (0.65, 0.84) | 0.025 | 86.9 | <0.001 |  |
|  | International | | 1 | 0.97 (0.92, 1.03) | 0.0 | - | - |  |
| Age | | |  |  |  |  |  | 0.471 |
|  | | <60 years | 8 | 0.73 (0.63; 0.85) | 0.034 | 91.6 | <0.001 |  |
|  | | ≥60 years | 4 | 0.84 (0.73; 0.98) | 0.018 | 89.3 | <0.001 |  |
| Duration of follow-up | | |  |  |  |  |  | 0.018 |
|  | <10 y | | 10 | 0.80 (0.72, 0.88) | 0.017 | 84.0 | <0.001 |  |
|  | ≥10 y | | 2 | 0.67 (0.30, 1.47) | 0.318 | 97.2 | <0.001 |  |
| Number of cases | | |  |  |  |  |  | 0.793 |
|  | <100 | | 2 | 0.72 (0.36, 1.45) | 0.020 | 83.0 | 0.015 |  |
|  | 100-<500 | | 9 | 0.77 (0.68, 0.87) | 0.024 | 90.5 | <0.001 |  |
|  | ≥500 | | 1 | 0.73 (0.66, 0.81) | - | - | - |  |
| Risk of bias | | |  |  |  |  |  | 0.346 |
|  | moderate | | 5 | 0.79 (0.68, 0.93) | 0.019 | 82.0 | <0.001 |  |
|  | serious | | 7 | 0.76 (0.66, 0.88) | 0.030 | 93.2 | <0.001 |  |
| * SHRs were calculated using random effects models. | | | | | | | | |
| † *p*-value for heterogeneity within subgroups. | | | | | | | | |
| ‡ *p*-value for heterogeneity between subgroups estimated using meta-regression | | | | | | | | |
| ^#^ one study did not provide information about menopausal status | | | | | | | | |

**Table S7.2.** Type 2 diabetes.

|  | | | **N of studies** | **SHRs**  **(95% CI)** * | ***Tau^2^*** | ***I^2^* (%)** | ***P*_within_ _subgroup_** † | ***P*_between_ _subgroup_** ‡ |
| --- | --- | --- | --- | --- | --- | --- | --- | --- |
| All studies | | | 6 | 0.96 (0.93, 0.99) | 0.001 | 71.8 | 0.003 | - |
| Assessment of physical activity | | |  |  |  |  |  | - |
|  | | questionnaire | 6 | 0.96 (0.93, 0.99) | 0.001 | 71.8 | 0.003 |  |
|  | | interview | - | - | - | - | - |  |
| Geographic area | | |  |  |  |  |  | 0.165 |
|  | Asia | | 1 | 0.68 (0.46, 1.01) | 0.0 | - | - |  |
|  | Europe | | 2 | 0.98 (0.96, 0.99) | 0.0 | 0.0 | 0.426 |  |
|  | USA | | 3 | 0.90 (0.81, 1.00) | 0.007 | 85.6 | 0.01 |  |
| Age | | |  |  |  |  |  | 0. 672 |
|  | | <60 years | 1 | 0.90 (0.83, 0.97) | 0.0 | - | - |  |
|  | | ≥60 years | 5 | 0.97 (0.94, 1.00) | 0.0004 | 69.6 | 0.011 |  |
| Duration of follow-up | | |  |  |  |  |  | 0.097 |
|  | <10 y | | 4 | 0.96 (0.93, 0.99) | 0.001 | 76.3 | 0.005 |  |
|  | ≥10 y | | 2 | 0.95 (0.86, 1.04) | 0.004 | 79.6 | 0.027 |  |
| Number of cases | | |  |  |  |  |  | 0.562 |
|  | <100 | | 1 | 0.68 (0.46, 1.01) | 0.0 | - | - |  |
|  | 100-<500 | | 1 | 0.90 (0.83, 0.97) | 0.0 | - | - |  |
|  | ≥500 | | 4 | 0.97 (0.95, 1.00) | 0.0003 | 69.7 | 0.019 |  |
| Risk of bias | | |  |  |  |  |  | 0.672 |
|  | moderate | | 1 | 0.90 (0.83, 0.97) | 0.0 | - | - |  |
|  | serious | | 5 | 0.97 (0.94, 1.00) | 0.0004 | 69.6 | 0.011 |  |
| * SHRs were calculated using random effects models. | | | | | | | | |
| † *p*-value for heterogeneity within subgroups. | | | | | | | | |
| ‡ *p*-value for heterogeneity between subgroups estimated using meta-regression | | | | | | | | |

**Table S7.3.** Ischemic heart diseases.

|  | | | **N of studies** | **SHRs**  **(95% CI)** * | ***Tau^2^*** | ***I^2^* (%)** | ***P*_within_ _subgroup_** † | ***P*_between_ _subgroup_** ‡ |
| --- | --- | --- | --- | --- | --- | --- | --- | --- |
| All studies | | | 8 | 0.88 (0.83, 0.93) | 0.003 | 86.5 | <0.001 | - |
| Assessment of physical activity | | |  |  |  |  |  | 0.244 |
|  | | questionnaire | 5 | 0.92 (0.86, 0.98) | 0.002 | 87.7 | <0.001 |  |
|  | | interview | 3 | 0.84 (0.78, 0.92) | 0.002 | 44.6 | 0.165 |  |
| Geographic area | | |  |  |  |  |  | 0.953 |
|  | Asia | | 2 | 0.76 (0.47, 1.23) | 0.105 | 93.2 | <0.001 |  |
|  | Europe | | 4 | 0.60 (0.41, 0.88) | 0.111 | 78.9 | 0.003 |  |
|  | USA | | 1 | 0.89 (0.82, 0.95) | 0.0 | - | - |  |
|  | International | | 1 | 0.96 (0.94, 0.98) | 0.0 | - | - |  |
| Age | | |  |  |  |  |  | 0. 417 |
|  | | <60 years | 2 | 0.76 (0.47, 1.23) | 0.112 | 93.2 | <0.001 |  |
|  | | ≥60 years | 6 | 0.83 (0.73, 0.93) | 0.013 | 85.9 | <0.001 |  |
| Duration of follow-up | | |  |  |  |  |  | 0.442 |
|  | <10 y | | 6 | 0.93 (0.88, 0.97) | 0.002 | 80.7 | <0.001 |  |
|  | ≥10 y | | 2 | 0.71 (0.51, 0.99) | 0.042 | 84.3 | 0.012 |  |
| Number of cases | | |  |  |  |  |  | 0.183 |
|  | <100 | | - | - | - | - | - |  |
|  | 100-<500 | | 5 | 0.61 (0.44, 0.85) | 0.101 | 84.4 | <0.001 |  |
|  | ≥500 | | 3 | 0.94 (0.90, 0.97) | 0.001 | 83.5 | 0.002 |  |
| Risk of bias | | |  |  |  |  |  | 0.957 |
|  | moderate | | 5 | 0.73 (0.61, 0.87) | 0.025 | 82.9 | <0.001 |  |
|  | serious | | 3 | 0.96 (0.93, 0.98) | 0.0002 | 54.5 | 0.111 |  |
| * SHRs were calculated using random effects models. | | | | | | | | |
| † *p*-value for heterogeneity within subgroups. | | | | | | | | |
| ‡ *p*-value for heterogeneity between subgroups estimated using meta-regression | | | | | | | | |

**Table S7.4.** Chronic obstructive pulmonary disease.

|  | | | **N of studies** | **SHRs**  **(95% CI)** * | ***Tau^2^*** | ***I^2^* (%)** | ***P*_within_ _subgroup_** † | ***P*_between_ _subgroup_** ‡ |
| --- | --- | --- | --- | --- | --- | --- | --- | --- |
| All studies | | | 2 | 0.70 (0.45, 1.10) | 0.099 | 94.0 | <0.001 | - |
| Assessment of physical activity | | |  |  |  |  |  | - |
|  | | questionnaire | 2 | 0.70 (0.45, 1.10) | 0.099 | 94.0 | <0.001 |  |
|  | | interview | - | - | - | - | - |  |
| Geographic area | | |  |  |  |  |  | - |
|  | Asia | | - | - | - | - | - |  |
|  | Europe | | 2 | 0.70 (0.45, 1.10) | 0.099 | 94.0 | <0.001 |  |
|  | USA | | - | - | - | - | - |  |
|  | International | | - | - | - | - | - |  |
| Age | | |  |  |  |  |  | - |
|  | | <60 years | 1 | 0.87 (0.82, 0.92) | - | - | - |  |
|  | | ≥60 years | 1 | 0.55 (0.44, 0.68) | - | - | - |  |
| Duration of follow-up | | |  |  |  |  |  | - |
|  | <10 y | | 1 | 0.55 (0.44, 0.68) | - | - | - |  |
|  | ≥10 y | | 1 | 0.87 (0.82, 0.92) | - | - | - |  |
| Number of cases | | |  |  |  |  |  | - |
|  | <500 | | - | - | - | - | - |  |
|  | ≥500 | | 2 | 0.70 (0.45, 1.10) | 0.099 | 94.0 | <0.001 |  |
| Risk of bias | | |  |  |  |  |  | - |
|  | moderate | | - | - | - | - | - |  |
|  | serious | | 2 | 0.70 (0.45, 1.10) | 0.099 | 94.0 | <0.001 |  |
| * SHRs were calculated using random effects models. | | | | | | | | |
| † *p*-value for heterogeneity within subgroups. | | | | | | | | |
| ‡ *p*-value for heterogeneity between subgroups estimated using meta-regression not possible because of small numbers of studies (n=2) | | | | | | | | |
